# Changes in agglomeration and productivity are poor predictors of inequality across the archaeological record

**DOI:** 10.1073/pnas.2400693122

**Published:** 2025-04-14

**Authors:** Scott G. Ortman, Amy Bogaard, Jessica Munson, Dan Lawrence, Adam S. Green, Gary M. Feinman, Shadreck Chirikure, Johannes H. Uhl, Stefan Leyk

**Affiliations:** ^a^Institute of Behavioral Science, University of Colorado Boulder, Boulder, CO 80309; ^b^Santa Fe Institute, Santa Fe, NM 87501; ^c^Department of Anthropology, University of Colorado Boulder, Boulder, CO 80309; ^d^School of Archaeology, University of Oxford, Oxford OX1 3TG, United Kingdom; ^e^Department of Anthropology-Sociology, Lycoming College, Williamsport, PA 17701; ^f^Department of Archaeology, Durham University, Durham DH1 3LE, United Kingdom; ^g^Department of Archaeology and Environment, University of York, York YO1 7EP, United Kingdom; ^h^Department of Geography, University of York, York YO1 7EP, United Kingdom; ^i^Negaunee Integrative Research Center, Field Museum of Natural History, Chicago, IL 60605; ^j^Joint Research Centre, European Commission, Ispra 21027, Italy; ^k^Department of Geography, University of Colorado Boulder, Boulder, CO 80309

**Keywords:** archaeology, urban science, real estate, economic development, inequality

## Abstract

We address three basic issues regarding the long-term dynamics of inequality in society. First, we consider the interpretation of residence sizes in socioeconomic terms by comparing statistical patterns extracted from the Global Dynamics of Inequality (GINI) Project database with those from the 21st-century United States. Second, we examine the degree to which the size and productivity of human networks is systematically related to inequality. Finally, we investigate relationships between productivity and productivity growth distributions for patterns of inequality and development across preindustrial societies. We find that across preindustrial societies residence size distributions provide a reasonable proxy for the distribution of productivity (income, a flow of physical and social resources to the group) and a minimum estimator for the distribution of wealth (a stock of such resources accumulated over time); that scale and productivity affect levels of inequality but account for only a small fraction of the observed variance across societies; and that inequality growth is independent of productivity growth, on average and over time. These findings have important implications for efforts to promote more equitable economic development in the present.

## Significance

Comparisons of archaeological and contemporary real estate data show that in preindustrial societies variation in residential building area is proportional to income inequality and provides a conservative estimator for wealth inequality. The archaeological record also shows that the most reliable way to promote equitable economic development is through policies and institutions that reduce the covariance of current household productivity with productivity growth. There are many examples of this pattern in the archaeological record.

The social sciences have long debated whether income inequality rises with increases in the scale and productivity of human societies over the long term (1–5). Within archaeology, residence size has come to be seen as a useful and widely available proxy for investigating such questions empirically and statistically (6–10). The papers in this Special Feature use statistical patterns in residence sizes to document the emergence of economic inequality, identify its fundamental drivers, and catalog variation in relationships between inequality and other social properties and processes, all with an eye toward expanding the contemporary relevance of archaeological evidence. A key issue in this chain of reasoning is the relationship between residence areas and other socioeconomic properties of households captured in contemporary statistics. This is a nontrivial issue given variation in the demographic composition of residences across and within societies (11), and technological, institutional, and scalar differences between preindustrial societies and those of the contemporary world (12–16).

Here, we first address the interpretation of residence sizes through comparison of statistical patterns observed in the contemporary and preindustrial worlds. Next, we examine the degree to which the size and productivity of human networks is associated with residential disparities in preindustrial societies. Finally, we consider the effect of a fundamental feature of the statistics of skewed and changing distributions—the covariance of residence size with the growth rate in residence size—to expose relationships between society-wide changes in inequality and productivity. The contemporary data we utilize include information on housing units by US metropolitan statistical area drawn from the Zillow Transaction and Assessment (ZTRAX) database [(17, 18), and *Materials and Methods*], and the archaeological data include information on residential buildings in the Global Dynamics of Inequality (GINI) Project database (see refs. 19 and 20 and *SI Appendix* on the structure, construction, and content of this database).

## Results

### Stocks, Flows, and Residence Sizes.

We begin with the basic distinction between income—a flow of resources to a household per unit time—vs. wealth—a stock of such resources that accumulates over a longer period. Income, a socioeconomic rate, is generated by the production of goods and/or services and is typically measured annually to correspond to cycles of harvests and/or taxation. Here, we use the more general term “productivity” as a synonym for income. Wealth, on the other hand, is a stock derived from saved resources. When these resources facilitate the collection of additional returns, they are often considered assets, or sometimes capital. Any cross-sectional assessment of wealth will capture fortunes accumulated over varying lengths of time. When measured at the household level, the resources involved in generating income and/or in accumulating wealth can be embodied in the capacities of individuals and in the number of residents who cooperate in production, material in the form of agricultural produce or manufactured goods, or social in the form of obligations receivable from others (21). Importantly, both income generation and wealth accumulation involve conversions of resources from more to less bulky material forms, and from embodied and material forms to social forms. Wealth and income are also related in that household wealth accumulates from surplus income, and a range of assets including but not limited to land, livestock, and real estate support the generation of additional income (8); but in general, wealth disparities are more pronounced than income disparities.

The primary observation compiled for the GINI Project is the enclosed/roofed area of a residential building, and one would expect households with more residents, larger incomes, and more accumulated wealth, to live in larger residences than households with fewer residents, smaller incomes, and less accumulated wealth. Given this premise, and our framing of individuals as embodied resources constituting a factor of household-level production, a key question is whether variation in residence area is more closely proportional to variation in income or to variation in wealth. Below, we examine this question using contemporary data from the ZTRAX database, and archaeological data from the GINI Project database. Our results suggest that in preindustrial societies residence areas provide a reasonable index of the income distribution, and a conservative or minimal index of the wealth distribution.

### Residence Size in the Contemporary World.

In contemporary industrialized nations the physical size of a residence is related to several factors, including the annual income and the net worth of the residents, relative to local costs. On the income side, the size of a house relates to the ability of the residents to pay monthly rent or a monthly mortgage; and on the wealth side, home equity is a primary component of net worth for many households. However, the relationship between residence size and household wealth is complex. Residential property values vary by location: land rents are higher in larger cities and near the centers of cities, so house prices per square meter are higher in these contexts as well (22–26). In addition, wealthier households often own multiple residences, while poorer households rent and do not own any residences at all. In the latter case, the tenants use their income to pay for their residential space, but the value of this space contributes to the net worth of the landlord, not the tenants.

Some of these effects can be quantified using data from ZTRAX and other sources. For example, Table 1 summarizes effects of settlement population size for residential properties in the contemporary US. Each row presents an ordinary least squares regression model of population size vs. an aggregate property of real estate across cities [metropolitan or core-based statistical areas (CBSA)]. The data are log-transformed prior to analysis, so the slope of the resulting fit line reflects the elasticity (relative increase) of the dependent variable relative to population size. A slope equal to one indicates that the dependent variable increases proportionately to population; a slope greater than one indicates that the dependent variable increases faster than population; and a slope less than one indicates that the dependent variable increases more slowly than population. These elasticities can be converted to per capita measures by subtracting one from the slope. The table shows that total wages increase faster than population, indicating that higher-paying jobs are more common in larger cities (Table 1, *A*). These higher incomes tend to cancel out higher housing costs in larger cities (24), such that the total number of residential buildings, and the total area of residential buildings, are both proportional to population across city sizes (Table 1, *B* and *C*). Contemporary residence areas thus do not track incomes (or wealth) at this level. Note, however, that lot sizes are systematically smaller in larger cities (their price per unit area increases faster than household incomes (27), Table 1, *D*); and property values increase with city population with a greater elasticity (β=1.22) than wages (β=1.14) consistent with interpretation of the former as a wealth indicator that increases faster than incomes (Table 1, *E*).

**Table 1.** Scaling of contemporary real estate measures with urban populations

Analysis	Independent	Dependent	Intercept (SE)	Slope (SE)	R^2^	F-statistic (df1, df2)	*P*-value	N
A	1969 to 2009 MSA Population^*^	1969 to 2009 MSA Wages (USD)		1.114 (0.025)	0.935			363
B	2010 CBSA population	Total residential property Count	−0.892 (0.246)	0.962 (0.021)	0.740	2,043 (1,718)	<2.2e-16	720
C	2010 CBSA population	Total area of residences (km^2^)	−9.709 (0.315)	1.017 (0.027)	0.660	1,396 (1,718)	<2.2e-16	720
D	2010 CBSA population	Total lot area (km^2^)	−1.143 (0.497)	0.598 (0.043)	0.213	193.9 (1,715)	<2.2e-16	717
E	2010 CBSA population	Total property value (USD)	7.184 (0.460)	1.224 (0.040)	0.569	947.6 (1,718)	<2.2e-16	720

^*^From (27: *SI Appendix*, Table S2, 28: figure 2).

In addition, property values, and by extension, land rents, tend to decrease with distance from the central business district (25, 26). In the standard monocentric urban model in economics, households are modeled as being “indifferent” as to their location, given a budget which balances income with housing and transport costs (22–24). Today, due to low transport costs, individuals can live at a considerable distance from the location where they generate their incomes, so they can afford to live in a larger residence in the suburbs than they could in the city center. The net result is that, as distance from a center increases within a commuting zone, residence values decrease per unit area, and residence sizes increase, until an inflection point is reached where transport costs outweigh the income gains from work in the city center. This pattern is shown for CBSA in the contemporary US in *SI Appendix*, Fig. S1. Overall, these patterns show that in the contemporary world residence areas do not provide a straightforward index of household incomes or wealth. Fortunately, the situation appears more straightforward in preindustrial contexts.

### Residence Size in Preindustrial Societies.

Some aspects of contemporary real estate are not obtainable from the archaeological record. As examples, information on the absolute or relative social or monetary values of residences is rarely preserved, and it is usually impossible to determine whether residents were owners or tenants of the building they occupied. As a result, it is not possible to assess property ownership or asset values using archaeological evidence.

Another substantial but surmountable difference between the past and present is the varied composition and social structure of residential groups. The GINI Project database includes a wide range of residence types, from Classic Maya plazuela groups to Iroquoian long houses, New Guinean men’s and women’s houses, Central Mexican apartment compounds, ancestral Pueblo unit pueblos, Roman villas, and Inka kanchas, among many others. In addition, many people who lived and worked in elite residences were workers and servants, not family members. We use the term residence to highlight that our units of measurement (and population) are residential buildings, which do not always correspond to households or families, and that our unit of analysis is the residential group which itself varied substantially across spatial and temporal contexts. We view such groups as basal units of production and consumption, within which resources were shared in some way, and with the number of people in the group representing the embodied component of its productivity.

The composition of residential groups varies substantially across preindustrial societies, but we find no evidence that household composition systematically affects residential disparities. *SI Appendix*, Fig. S2 summarizes Gini coefficients of residence area across settlements from small-scale societies (family-level, local, big-man, and simple chiefdom, following ref. 29), where variation in household composition is most prevalent. These summaries show that residence sizes are generally larger but not necessarily more disparate in societies where residential groups consist of extended or multiple families.

**Fig. 1.**
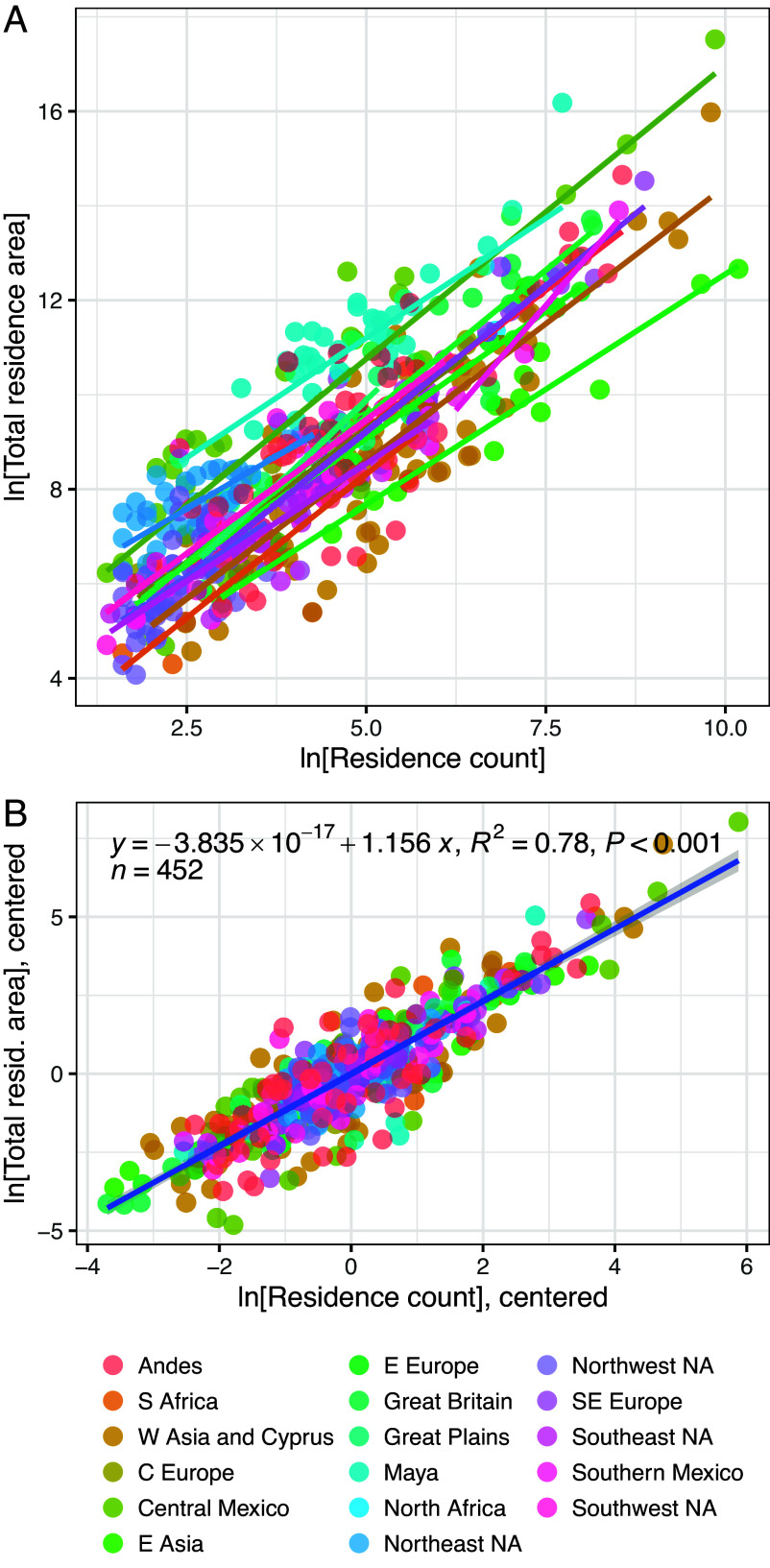
Ordinary least-squares regression of log-transformed residence counts and total residence areas for individual settlements, by region. Note that in (*A*) the slopes of the relationships are broadly similar, but the intercepts, which reflect baseline productivity, vary by region; and in (*B*), the data are centered to remove variation in the intercepts before conducting a pooled analysis. The resulting slope is within two decimal places of the expected value for a socioeconomic rate, as discussed in the main text.

We also find three patterns which are consistent with an interpretation of residence area as an index of residential group productivity. The first pattern is the scaling of total residence area with total residence count. In contemporary data, both measures scale proportionately with population (Table 1, *B* and *C*), but in the archaeological record, total residence count is proportional to population while total residence area scales with total residence count as a socioeconomic rate, with a similar elasticity to urban incomes. This pattern is shown for sedentary societies in the GINI Project database in Fig. 1. Each point represents a settlement where (log-transformed) total residence count (population) and total residence area (housing consumption) have been extrapolated from the observed area (the “window” area) to the total settlement area, based on the assumption that the observed portion provides a reasonable sample of the whole (see *materials and methods*, below). These data include the areas of palaces and other elite residences where a range of administrative functions, production, and storage occurred. Fig. 1*A* shows the relationship between these two measures, by region (mobile hunter-gatherer societies are excluded; see the *Materials and Methods*). This analysis shows that, while the intercept representing the average area of an isolated residence varies across regions, most regions show similar slopes (also see *SI Appendix*, Table S1). In Fig. 1*B* we control for regional variation by subtracting the mean values for the corresponding region and period from each data point. This has the effect of “centering” the data, such that all data series have the same center point at (0,0). This allows one to estimate the common slope across data series using a larger sample size. Based on models from urban science (see refs. 24, 27, 30, and 31, and references therein for derivations), the expected slope (β) for the scaling of an aggregate socioeconomic rate (such as GDP, wages, incomes, or interactions) with settlement population is β=1.167. The observed slope for total residence area across the archaeological record is β = 1.156 ± 0.029, within two decimal places and one SE of this expected value. This result is consistent with previous studies (32) which have consistently found that in sedentary preindustrial societies total residence area scales with residence count as a socioeconomic rate. Note also that in contemporary data the observed slope for total residence value (a measure of wealth) is much steeper than the observed slope for socioeconomic rates (Table 1, *A*). These results suggest that in preindustrial contexts residence area does not scale proportionately with population or residence value, but it does scale proportionately with other socioeconomic rates, such as income.

The second pattern is that residence areas decrease with distance from the center. Today, houses in urban agglomerations get larger on average with distance, to a certain point (*SI Appendix*, Fig. S1), due to relatively fast and low-cost commuting, which makes it feasible for households to generate incomes in the urban center but spend them in suburban areas, where land rents and other costs are lower. In contrast, transport was much slower and more energetically costly in the preindustrial world, so most households seem to have generated their incomes close to or within their residences. Even in Romano-British towns, a common type of residence was a rectangular building with its narrow dimension facing a street, and with a shop in the front and a living room in the back (28, 33). One would expect this difference to exert a systematic effect on the relationship between residence area and distance to central places.

**Fig. 2.**
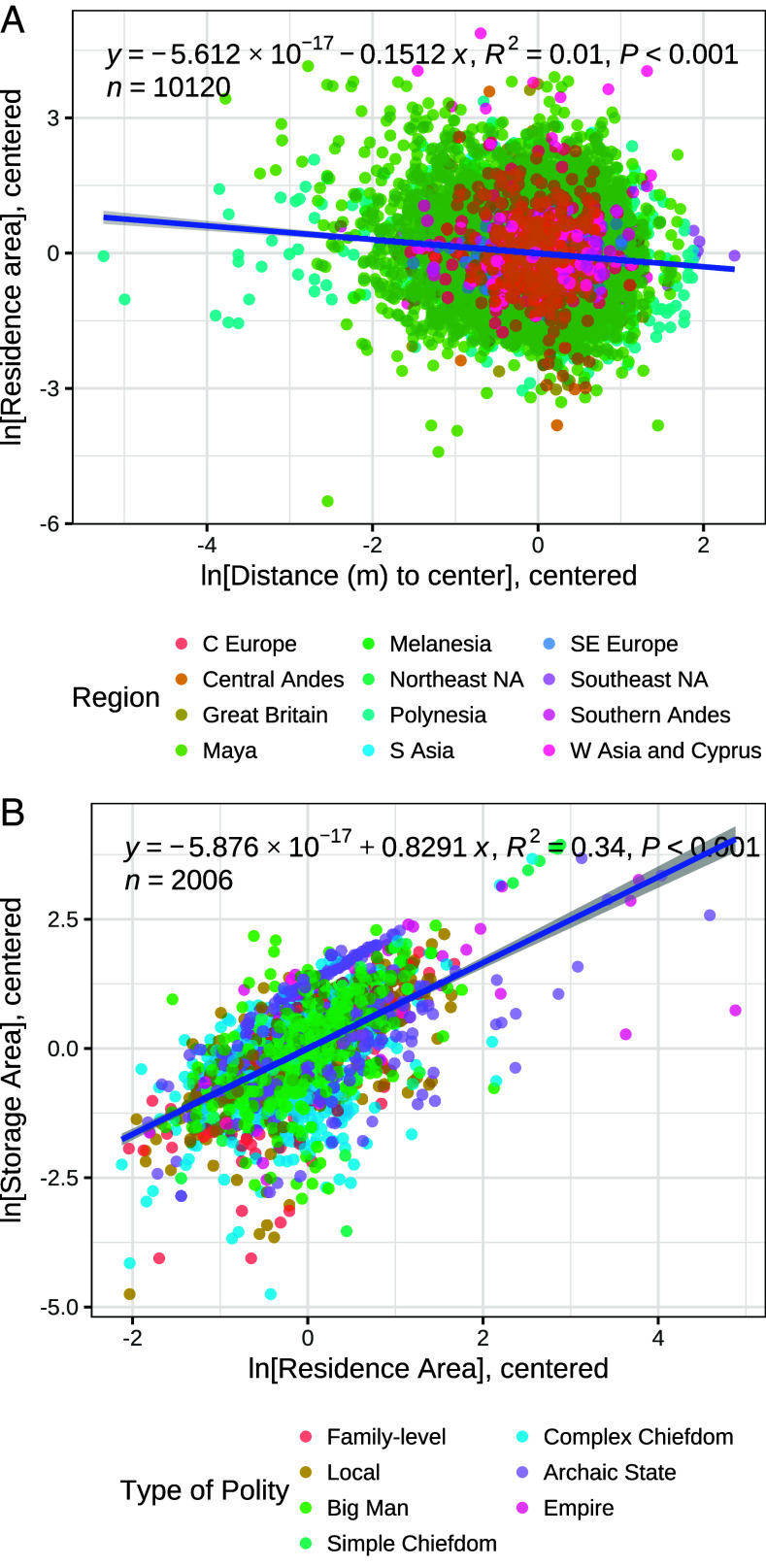
Ordinary least-squared regressions of log-transformed properties of individual residences: (*A*) residence area vs. distance from its settlement center; note that on average residence areas become slightly smaller with distance from the center; (*B*) storage area vs. residence area; note that storage takes up a decreasing fraction of the residence area as residence area grows. In both plots, the data are centered by region, but in (*B*) residences are color-coded by polity type to illustrate that the sublinear pattern transcends political organizations.

During data collection, when feasible, analysts recorded the straight-line distance of a given residential building from the center of its associated settlement (*Materials and Methods*). The relationship between distance and residence area is shown in Fig. 2*A*, with the data once again centered by region and period. The plot shows that residences decrease in physical size, on average, with distance from settlement centers. Although only a small fraction of the total variation in residence area is explained by distance, the relationship is significant and the effect is nontrivial. This is consistent with a model in which household productivity, in the general sense of the production of socioeconomic value, was higher closer to settlement centers, where most people engaged in social (nonagricultural) production close to or within their residences, and where residence areas reflect the outcomes of these activities. The only settlements with substantial data for which we observe the typical modern relationship are the Imperial Roman towns of Pompeii and Ostia (see *Materials and Methods* and *SI Appendix*, Fig. S3).

Finally, the third pattern is that areas set aside for storage increase more slowly than overall residence area. In preindustrial societies, a large fraction of overall production was of physical things that took up space, and these inventories needed to be stored somewhere until they were consumed or exchanged.

During data compilation, when feasible, analysts distinguished storage areas of residences based on the interpretations of the original recorders or contextual understanding of the archaeological remains. In some cases, storage areas are distinct architectural spaces; in others, they are storage pits with measured volumes; and in still others, recorders considered the ground floor of two-story buildings as storage area. If residence area increased proportionately to accumulated material wealth, one would expect an increasing fraction of this area to be devoted to storage. The GINI Project data demonstrate that this does not occur. Fig. 2*B* illustrates the relationship between these storage areas/volumes and residence areas. The data are once again centered by region to control for regional variation in the relationship due to technology, resources, and geography. However, in Fig. 2*B*, residences are color coded according to the type of polity in which they occur, following (29). The fit line indicates that, across societies and polity types, storage makes up a decreasing fraction of the residence area as residence area becomes larger (Fits for individual regions are given in *SI Appendix*, Table S2).

We interpret this pattern as an indication that wealth accumulation involved conversion of physical surplus into money, preciosities with high exchange value to volume ratios, or social obligations receivable through gifts and loans. Such practices are widespread in preindustrial societies, ranging from potlaches in the Northwest Coast of North America to competitive feasting in Melanesia, communal giveaways in the New Mexico Pueblos, corn beer fiestas in the Andes, and conversion of goods and services to coin in the Roman World (34–36). Because these stores of value take up much less space than primary production, a residence only needs to expand proportionately to the volume of people and things that flow through the house per unit time, including social groups that are hosted by the residents; it does not need to expand proportionately to the accumulating surplus value that results from these activities. This feature of the spatial requirements of different resources in turn reinforces the use of residence areas to index income distributions. Note also that centralized food storage involves removal of a fraction of agricultural production from residences, but so long as this fraction does not covary with residential outputs (as occurs with progressive taxation) its removal would not affect the observed scaling. Finally, this result suggests that, as household income increases, an increasing fraction of this income takes the form of information, services, and/or value-added goods that are more valuable per unit volume than agricultural staples.

Taken together, these patterns provide multiple lines of evidence that in nonindustrial societies residence areas are proportional to the income or productivity of residential groups, while providing only a minimal estimator of their accumulated wealth. The inability to track ownership groups archaeologically reinforces our conclusion that residential building size distributions are conservative relative to wealth distributions. This is because wealthy individuals could control multiple residential properties, but most households utilized only one residence at a time, regardless of whether they were owners or tenants. These findings also emphasize that residence areas index household incomes more directly and consistently in preindustrial contexts than they do in contemporary contexts. This is encouraging with respect to connecting the results discussed below, and in other papers of this Special Feature, to contemporary concerns. It is nevertheless important to reiterate that, even in preindustrial societies, a significant portion of a household’s net worth was reflected in the value of the residents’ social relationships, broadly construed, and socializing in the home was one of the means through which such value was generated and maintained. This social network value also contributed to household income and is reflected in residence size.

### The Association of Scale and Productivity with Inequality.

We now consider some of the basic factors argued to affect economic inequality in the literature (1–5). We focus here on information aggregated by settlement, region, and temporal phase; see refs. 37–39 for spatial analyses, for analyses at the intrasettlement level, and for comparisons of site vs. regional patterns. Socioeconomic rates–including wages, GDP, social media contacts, infectious disease rates, crime rates, and phone calls–commonly follow log-normal distributions, meaning that, while the distribution of the raw measure is skewed and has a long upper tail, the distribution of the log-transformed measure is approximately normal (40). Across the GINI Project database, the distributions of log-transformed residence areas, standardized by period and region, are also approximately log-normal (*SI Appendix*, Figs. S4 and S5). This feature implies that residential group incomes resulted from the multiplication of several independent variables (e.g., number of residents x hours of work per person x land productivity per ha x social contacts per person, etc.) (41). The Gini coefficient is also strongly correlated with the SD of log-normally distributed values (indeed, across the GINI Project database, the Pearson correlation between these two measures, calculated by region and period, is r = 0.9). This relationship implies that any factor that affects the SD of a log-transformed measure will also affect the Gini coefficient of that same measure. One such factor is the mean-log of *settlement population*, due to the relationship between sample size and dispersion, for small sample sizes. A second is the mean-log of residence area, which indexes the *average productivity* of residential groups due to the relationship between mean and variance in a normal distribution. Finally, a third factor is the intercept of the scaling relation between settlement population and productivity discussed above. This measure controls for the effects of scale for residential group productivity (Fig. 1) and represents the *baseline productivity* of a residential group working in isolation, or absent agglomeration effects (42). The intercept of the scaling relationship between population and productivity integrates household organization, transport technology, environmental variation, and energy-capture technology, while controlling for the effect of settlement population (Fig. 1). As discussed above, several studies have demonstrated that settlement population exerts a systematic effect on socioeconomic rates (*Materials and Methods*) (27, 30–32, 43).

**Fig. 3.**
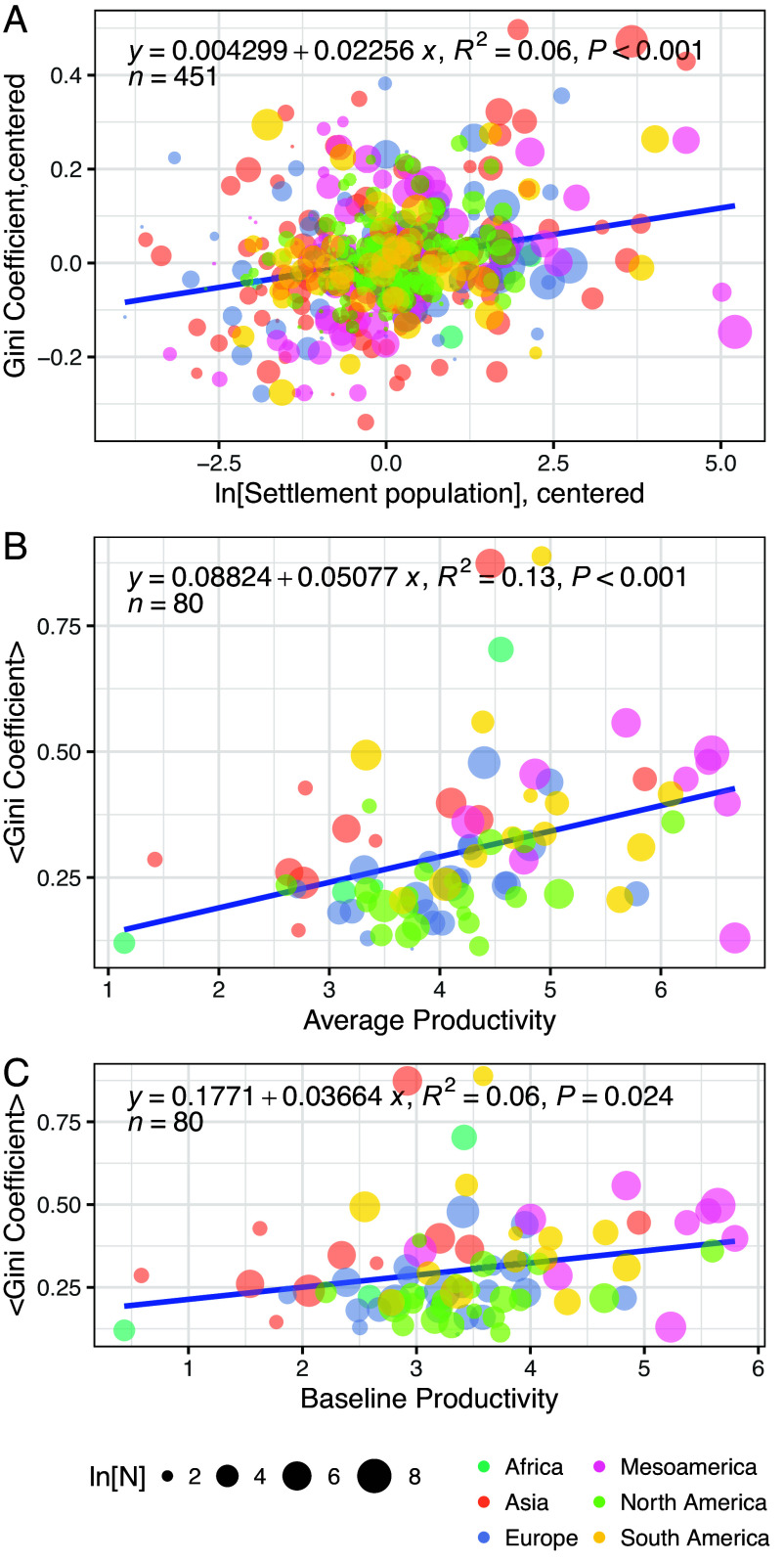
Effect of settlement population size, average productivity, and baseline productivity for levels of inequality. In (*A*) points represent individual settlements and are centered by region; and in (*B*) and (*C*) points represent averages across settlements from each region and phase. Symbol sizes are indexed to the number of measured residences for each group in each plot. The fits in all three panels are from ordinary least-squares regression.

Fig. 3 illustrates the effect of settlement population, average productivity, and baseline productivity for income inequality across the GINI Project database (*Materials and Methods*). Population agglomeration and productivity have both been cited as intrinsic drivers of increasing economic inequality in previous literature (1–5). The figure shows that all three factors are associated with rising income inequality, but most of the observed variation is left unexplained by these factors. The relationships for average and baseline productivity are similar because preindustrial settlements are relatively small in comparison to contemporary cities, so the scale effect is modest but still apparent. These results suggest that in the preindustrial world larger communities, and more productive societies, did exhibit higher levels of income inequality on average, despite recent claims to the contrary (44). It seems likely that these relationships reflect intrinsic constraints on the properties of human networks embedded in space in settlements, in that inequality is connected to heterogeneity and diversity, and both market size (population) and the division of labor (productive diversity) contribute to group-level productivity (45, 46). Nevertheless, most of the observed variation in income inequality is NOT accounted for by scale and productivity alone. It is also important to recognize that distributional changes of many different types (immiseration of the poor, elevation of the elite, etc.) can have the same effect on aggregate measures of inequality like the Gini coefficient. These results thus call for a deeper analysis.

### Effect of Distributed Growth.

Inequality is a statistical and distributional property of human networks that emerges and is managed over time, so the most fundamental driver of increasing income inequality must be the extent to which increases in income (and/or costs) are connected to existing income and/or wealth, at the level of the individual residential group. Anything that leads to a positive covariance between current income and growth in income will increase the level of income inequality over time; and anything that leads to a negative covariance will reduce it (46). The ideal data for investigating this phenomenon would be measurements of residences that can be associated with specific lineages across multiple generations. This is rarely available in the archaeological record and is not captured in the GINI Project database. The alternative, explored here, involves analysis of residence area distributions across time steps within specific regions (*SI Appendix*, Fig. S6). The key metric we examine here is the covariance of residence area with growth in residence area, by decile, across phases and within regions (*Materials and Methods*).

**Fig. 4.**
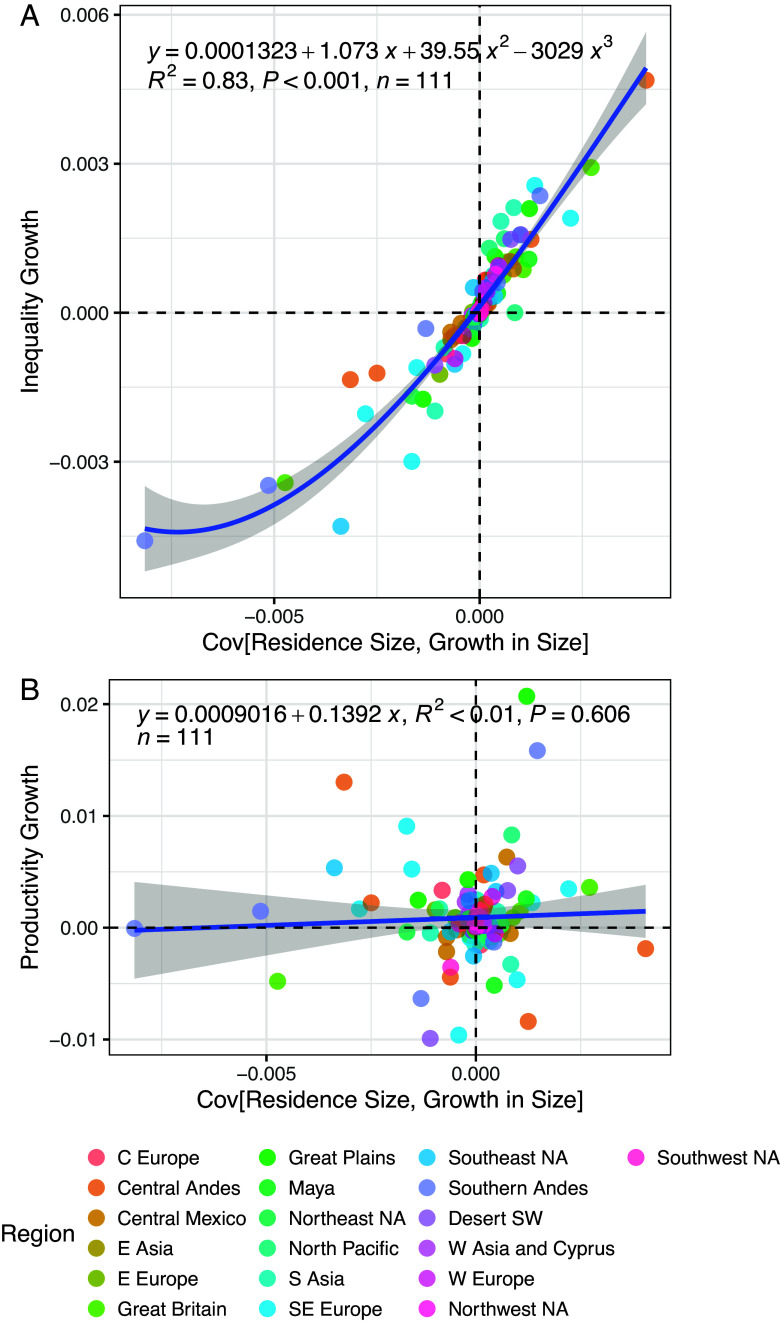
Relationship between the covariance of residence area and growth in residence area with (*A*) growth in inequality (cubic fit) and (*B*) overall growth in residence area (linear fit). Each symbol represents a region and phase.

Fig. 4 presents the results, with each point representing a region and phase. Fig. 4*A* shows that there is a strong relationship between the covariance of residence area and growth in residence area on the one hand, and overall inequality growth (change in the SD of log-transformed residence area) on the other. Essentially none of our cases show a positive covariance combined with a reduction in inequality, or a negative covariance combined with an increase. This is a straightforward byproduct of the statistics of distributed growth, and this is precisely the point. The societies reflected in Fig. 4*A* were organized at different scales, took root in different environments, and had widely varying technologies and institutions. But despite this variation, all follow the same statistical relationship. Fig. 4*B* further shows that there is no relationship between the covariance of residence size and growth vs. regional productivity growth. If these data tracked individual lineages over time, one would expect regional productivity growth to correlate with the covariance of income and growth at the lineage level. Unfortunately, these data only track the relative fortunes of residential groups by decile, with no tracking of social mobility between deciles across time steps. Nevertheless, the data show that the degree to which “the rich get richer” (as distinguished from “richer families get richer”) has no impact on changes in average societal living standards, as measured by the mean-log of residence area at the regional level. In other words, the processes that lead to improvements in residence group productivity appear to be separable from the most fundamental process that increases or decreases income inequality. *SI Appendix*, Fig. S7 plots productivity growth vs. inequality growth across time steps within regional sequences, with points labeled by the initial phase of each transition. This plot identifies a number of contexts where living standards improved and inequality declined at the same time, including Pueblo I in the North American Southwest, Lohman, and Early Mississippian in the American Bottom, Nebraska Phase, and Initial Middle Missouri in the North American Great Plains, the Late Preclassic Maya, Cucuteni B in Ukraine, Late Intermediate Central Andes, and Archaic Greece. Scholars have previously noted this combination for Archaic Greece in particular and have attributed it to the development of inclusive social institutions (10, 47, 48). It would be worthwhile to examine these other cases through a similar lens (see ref. 49).

## Summary

We find evidence that, in at least one *industrial* society, residence area is a poor proxy for household income or wealth. In contrast, we find statistical patterns in the scaling, spatial distributions, and storage allocations of *preindustrial* residential buildings which suggest that, across such societies, residence area is a reasonable proxy for household income or productivity, and provides a minimal estimator for accumulated wealth. Several of the complications that make it difficult to equate residence areas with household wealth or income today do not seem to have operated in the preindustrial past. Most notably, transport was much slower and more costly, leading to more straightforward relationships between residence location, residence size, and residential group productivity. This is exciting for archaeologists because it reinforces the value of residence area as a systematic economic indicator. Importantly, house area is inclusive of the extent to which the labor product of some households was appropriated by others due to their position in socio-political-economic networks. We also find that the population size and productivity of human networks both influence levels of economic inequality, but they only account for a small fraction of the observed variation across societies. Clearly, other factors can override the effects of scale and productivity for net levels of economic inequality.

Finally, we show that the covariance of residence area (as a proxy for income) with growth in residence area (growth in income) exerts a strong and systematic effect on regional-scale inequality but has no effect on regional-scale productivity. Overall, our results suggest that, while there is a modest effect of scale and productivity for inequality, there is no necessary relationship between productivity growth and inequality growth. Some degree of inequality growth is unavoidable as human networks become larger, more heterogeneous, and more interconnected. But factors influencing the covariance of income with income growth at the residential group level seem to exert a much stronger and more systematic effect. These findings suggest economic, environmental, technological, and institutional factors that lead to increasing inequality are agnostic with regard to increasing productivity. It also suggests it is possible for societies to reduce inequality and promote aggregate productivity at the same time. This is an exciting prospect for the present and an important area for further investigation (see ref. 46).

## Materials and Methods

### Use of Log-Transformed Measures.

The analyses in this paper utilize log-transformed measures, for two reasons. First, many social measures are log-normally distributed. Because of this, raw social data are highly skewed and are not appropriate for analysis methods, including regression, that assume normally distributed data. *SI Appendix*, Fig. S4 demonstrates that log-transformed residence areas are approximately normal and thus suitable for linear modeling. Second, log-transformation is involved in analyzing exponential growth because $P_{t}=P_{0}e^{\mathrm{rt}}$ and $\ln P_{t}=\ln P_{0}+rt$ are equivalent expressions. As a result, working with log-transformed measures yields results that are more readily interpreted in terms of growth rates.

### Contemporary United States.

We utilized data from the ZTRAX, a large, proprietary real-estate database covering most of the United States. ZTRAX is the result of collecting, integrating, and harmonizing cadastral and tax assessment data from official administrative source across all U.S. counties, and has been widely used by researchers across disciplines (17). The dataset contains attributes for over 150 million properties (18) and enables insights regarding historical and contemporary settlement patterns (50). We partitioned the ZTRAX dataset by US county and used a lookup table (51) to reallocate the records to their respective CBSA (52). CBSAs nest within US counties and delineate metropolitan areas based on their economic impact, commuting patterns, and other measures. Within each CBSA, we aggregated various attributes for residential properties (i.e., number of properties, total indoor area, total lot area, and total property value), representing units of analysis and encoded by the associated FIPS code. We utilized data from 2010 to describe the contemporary setting; data for earlier time slices would have to be retrodicted from the attributes associated with properties in the 2010 dataset. To assess relationships between city population size and distance to the city center, we grouped all single-family residential properties in ZTRAX by their respective CBSA and calculated for each CBSA the barycenter of properties (i.e., the mean latitude and longitude of all properties, referred to as “city center” hereafter). We then calculated the Euclidean distance of each single-family residential property to the city center and used a decile-based classification scheme to group the properties of each CBSA into ten distance bands. Moreover, we scaled the property size (i.e., the total indoor area of each residence) into the range [0,1], to account for potential regional variation in property size and calculated the median property size per distance band and CBSA. We then visualized the distributions of residence size over relative distance in *SI Appendix*, Fig. S1.

### Scaling Analysis.

An important issue in scaling analysis is the definition of spatial units, as such definitions have been shown to affect the coefficients of scaling relationships in contemporary data (53). According to settlement scaling theory, the appropriate units are areas within which residents mix socially across the space on a regular basis (27). For the contemporary USA, Metropolitan Statistical Areas defined by the Bureau of Economic Analysis are appropriate because they incorporate cross-county commuter flows. For preindustrial societies, in contrast, individual settlements defined archaeologically by concentrations of residences, infrastructure, and discarded artifacts are most appropriate because these encapsulate zones of daily pedestrian movement (30).

To produce the data analyzed in Fig. 1, we estimated the total number of residences and total square meters taken up by residences, for each phase of occupation at each site in the GINI Project database associated with the required data. In all analyses, the unit of population is the residential building. If a settlement is associated with a total residence count, this was used as the population estimate. If total residence count is not available, the number of residences within the area of observation (the window) is multiplied by the ratio of total site area to window area to estimate the total residence count. If neither is available, the settlement is excluded. In all cases, the log of total residence area is estimated as the mean-log residence area plus the log of residence count. When sites are subdivided more granularly than the chronological phase, we averaged the data for these subdivisions at the phase level.

We also made several decisions to account for idiosyncrasies in the data. For settlements from Central Mexico, most populations are estimated based on site area and surface artifact density, but a subset is estimated based on architectural remains. We filtered the data to include only the latter group for the scaling analysis, following earlier work (31). For Maya sites, the window areas are large, but site boundaries are often unknown, so we treated the window area as equivalent to the site area. We also excluded regions and periods characterized by mobile hunting and gathering due to the expectation, from previous studies of ethnographic data, that these societies would exhibit a distinct scaling from more sedentary societies (54). We excluded settlements from South Asia due to limited variation in the aggregate properties of settlements from this region, and from Polynesia due to the extremely dispersed nature of these settlements. Finally, in some cases, we combined sparsely sampled but adjacent regions to create larger subsamples, including Central Andes and Southern Andes; East Africa, Horn of Africa, and South Africa; Central Asia, Western Asia and Cyprus; and Western Europe and Central Europe. Readers interested in replicating this analysis can consult the R script (*Data, Materials, and Software Availability*).

The resulting estimates of total residence count and total residence area, for each site and phase, were then centered by region to produce Fig. 1*B*. One would ideally center the data by phase as well as region to capture changes in baseline residence area over time, but this was not done because it would have reduced sample sizes for many groups below a practical limit. Previous studies have shown that baseline residence sizes were consistent across long spans of time in several world regions but vary across geographies (32), so we sought to capture the time averaged relationship between residence count and total residence area by region.

### Residence Size vs. Distance to Center.

Settlement centers are modeled as a significant public open space, public building, or major crossroads, and distances were calculated as straight lines (see *SI Appendix* for more detail). *SI Appendix*, Table S3 lists the settlements for which this information was recorded. The data are centered by region and period to control for variation in transportation speed and cost across societies. Pompeii and Ostia are notable exceptions to the general pattern of decreasing residence size with distance (*SI Appendix*, Fig. S3), so we have excluded these sites from the analysis in Fig. 3. It is possible that modern-style real estate markets land-rent pressure began to take hold in core areas of the Roman Empire (but notably, not in the peripheral province of Britannia, which is well represented in the database).

### Storage Area vs. Residence Area.

Storage areas were recorded for about four percent of residences in the GINI Project database (see *SI Appendix* for more detail). Such space can only be distinguished in certain regional contexts, but these encompass a range of polity types and residence size distributions. Also, when it is possible to isolate storage space it can be measured consistently based on correlations between excavation results and plan maps. Compilers deferred to local expertise in all cases. When it is possible to distinguish storage space, but none is present in a residential building, the storage area is recorded as a zero; but when the presence or absence of storage space cannot be determined from the available information, it is treated as missing data. There are also archaeological records where storage space occurs, but it cannot be clearly associated with specific residences; such cases are excluded from this analysis. The data are centered by region to control for differences in baseline storage practices. The linear feature shown for a subset of residences in Fig. 2*B* derives from two-story dwellings where the ground floor was dedicated to storage. *SI Appendix*, Table S2 presents regression results for residence area vs. storage area by region.

### Scale and Inequality.

This analysis utilized the same dataset as the scaling analysis, discussed above. In Fig. 3*A* the residence count and Gini coefficient are calculated for each settlement and then centered by region; in Fig. 3*B* average productivity is calculated as $\frac{\left\langle lnY_{i}\right\rangle }{\left\langle lnN_{i}\right\rangle }$, the mean-log of total residence area divided by the mean-log of total residence count, across settlements from a given region and period; and in Fig. 3*C* baseline productivity $Y_{0}$ is calculated from the center of the data for each region and period using the standard scaling relation, $lnY_{0}=\left\langle lnY_{i}\right\rangle -\frac{7}{6}*\left\langle lnN_{i}\right\rangle$; see ref. 46.

### Covariance of Residence Size and Growth.

We grouped residence areas by region and phase and calculated the mean-log residence area for each decile of the distribution for each group. We then calculated the growth in this value, by decile, from one phase to the next. We used these two series of values to calculate the covariance of residence area with growth in residence area for each region and phase transition. Finally, we compared this covariance to the overall growth in inequality (SD of log-transformed residence areas) and overall growth in productivity (mean of log-transformed residence areas) across phase transitions at the regional scale. In this case, we used the SD of log-transformed residence areas, and not the Gini coefficient, due to the former’s more direct connection with growth accounting in economics. For growth calculations, the elapsed time is based on the midpoint date of sites assigned to each phase.

We removed a few problematic groups: General Mohenjo-Daro, due to overlapping span with other, more precisely defined phases from South Asia; South African sites due to sparse data overall; Fremont sites from the US Southwest due to the fact that this tradition is much less precisely dated than the others from this region; Chan Chan from the Central Andean sequence due to oversampling of elite residences; and Navajo sites from the US Southwest because these represent a separate cultural tradition from other communities in the region, and this tradition has limited time depth. Finally, we performed a cubic fit in Fig. 4*A* because it produced a closer fit to the distribution of points than a linear fit.

## Supplementary Material

Appendix 01 (PDF)

## Data Availability

All scripts and data for replicating the analyses and reproducing main and supplementary figures are provided as an R script and associated data files at tDAR (55) and Figshare (56). Project collaborators continue to identify and correct minor data entry errors in the project database, so individuals seeking to replicate results in this paper using the archived data and scripts may notice minor discrepancies in statistical outputs.
