# Supplementary material for: Changes in agglomeration and productivity are poor predictors of inequality across the archaeological record
Source: Proc Natl Acad Sci U S A. 2025 Apr 14;122(16):e2400693122. doi: 10.1073/pnas.2400693122 (PMC12037055; doi:10.1073/pnas.2400693122)
Supplement: Supplementary file 1 — Appendix 01 (PDF) [file pnas.2400693122.sapp.pdf]

PNAS

Supporting Information for

Changes in Agglomeration and Productivity are Poor Predictors of Inequality Across the Archaeological Record

Scott G. Ortman, Amy Bogaard, Jessica Munson, Dan Lawrence, Adam Green, Gary Feinman, Shadreck Chirikure, Johannes H. Uhl, Stefan Leyk

Scott G. Ortman
Email: scott.ortman@colorado.edu

This PDF file includes:

Supplementary Text
Figures S1 to S8
Tables S1 to S3
References Cited

Supplementary Text

The GINI Project Database

The data analyzed in this paper derive from a relational database compiled over three years (2021-2024) for the Global Dynamics of Inequality (GINI) project. The structure of the database was defined by the core collaboration team at the project kickoff meeting in November 2021 and contains summary information for around four thousand settlements and about fifty-three thousand residential buildings from those settlements. Data compilation was more opportunistic than systematic, focusing on archaeological records of preindustrial societies known primarily through their material remains, although some data from ethnographically documented small-scale societies were also included. Data compilation focused on times and places where traces of residential buildings are consistently preserved and can be defined and measured through surface survey, excavation, or remote sensing, including lidar. The resulting database contains information for archaeological sites from the US Southwest, Midwest, Southeast, Northeast and Northwest Coast of North America; the Central and Southern Andes in South America; Britain, Central, Eastern, and Southeastern Europe; Western, Central, Southern, and East Asia; Hawaii, New Zealand, Easter Island, and New Guinea in Oceania; and Northern, Western, Eastern, and Southern Africa. The oldest settlements in the database are from more than 20kyBP, and the most recent date from the mid-20th century, but most of them date from 12 to 1.5 kyBP in the Eastern Hemisphere, and from 7.5 to .3 kyBP in the Western Hemisphere (1).

The basic unit of measurement is a **residential building**, which need not correspond to a household or family, and our basic unit of analysis is the residential group which itself varied substantially across spatial and temporal contexts. Across the archaeological record there is substantial variation in the arrangements of rooms and buildings into residences, and equally substantial variation in the social groups that occupied these spaces. A few examples of this variation, and how we handled it, follow:

- Maya plazuela groups, several buildings arranged around a courtyard, treated as a single residence with multiple rooms

- Teotihuacan Apartment Compounds, a compound containing several residences, treated as a single residence for a large kin group

- Inka kanchas, several buildings connected by low walls surrounding a courtyard, treated as a single residence with multiple rooms

- Ancestral Pueblo unit pueblos, a small aboveground roomblock and adjacent subterranean pit structure, treated as a single residence with multiple rooms

- Hohokam courtyard groups, several residential pit structures arranged around a courtyard, treated as a single residence with multiple rooms

- Iroquoian long houses, a long building containing multiple residential areas, treated as a single residence for a large kin group

- Northwest Coast plank houses, a long building containing multiple residential areas, treated as a single residence for a large kin group

- Roman villas, several buildings clustered close together, each building treated as a separate residence

- Roman Insulae, multi-story apartment buildings along streets, subdivided into residences based on access patterns

- Iron age round houses, round buildings, sometimes clustered within enclosures, each building treated as a separate residence

- New Guinea men's and women's houses, known through ethnographic observation, each treated as a separate residence

- Chinese Neolithic houses, surface structures clustered into sectors, each structure treated as a separate residence

- Harappan compounds, groups of rooms and courtyards defined by access patterns, treated as single residences.

Our goal in defining "residential buildings" was to isolate basal units of production and consumption, within which resources were shared in some way, and with the number of people in the group representing the embodied component of its productivity.

A basic issue the team confronted was how to compile consistent information given variation in housing in past societies, and variation in preservation, archaeological research traditions, and data collection methods across world regions. The core collaboration team chose to default to regional expertise and data collection methods in all cases and defined database variables with this variation in mind. In most cases we compiled information from the published literature and/or archival data sources, but in some cases, contributors shared results of new, unpublished fieldwork. The raw data file analyzed in this paper, and the processed data files analyzed in other contributions to this Special Feature, are available for download at: https://core.tdar.org/project/496853/the-global-dynamics-of-inequality-gini-project.

Data collected for each "site" include the following:

Bigregion, Region, Subregion, Subarea—a series of nested geographical groupings into which sites and associated residences can be placed

SitePeriod, SitePhase—nested culture/historical groupings defined by research traditions in each region. In combination with the geographical groupings above, these fields provide a basis for aggregating information in a variety of ways above the settlement level.

SiteID, SiteName—the site number and the name by which the given settlement is known in the local literature. For some regions each "site" record represents a specific layer of occupation at a settlement (e.g., Western Asia), whereas in others it represents the entire occupation (e.g., Roman Britain), a specific chronological period (e.g. Central Mexico), or a portion of a settlement (e.g., South Asia). This is an unavoidable consequence of the varying ways source information is organized across archaeological research traditions. Thus, the GINI Project uses the term "site" to refer to a combination of location and time. Individual buildings are also assigned to periods and phases (see below).

BeginSite, EndSite, BeginLocation, EndLocation (BCE/CE)— beginning and ending date of the associated site and its other attributes and buildings; and the beginning and ending dates representing the period of continuous occupation at that location. The second set of dates can be longer than the first. BCE dates are negative. Because the chronological relations between individual residences and sites vary across the database, we also captured the beginning and ending date of each residential building (see below). For some regions these duplicate the site-level dates, but in others they are distinct and generally more precise.

Latitude, Longitude (Decimal Degrees)—coordinates of a central point within the site, truncated to two decimal points to mask precise site locations.

NOfLevels, WhichLevel—the position of the site within the regional settlement hierarchy. Across the database, entries range from 1 to 6 and are missing for only a few dozen of more than 4,000 site records. In general, larger and more complex polities have more levels.

TypeOfPolity, PolityPop, PolityArea, PolityEcon, PolityGov, PolityArch—a series of variables that capture information on the type of polity the site is associated with. Polity type follows the classification in (1), and polity population and area estimates are taken from the literature when available. Contributors also rated the economy, government, and architecture of each polity as being relatively collectively or autocratically-organized, following the scheme developed in (2).

Fortified?—whether or not the site exhibits defensive architecture or setting.

TotalArea, WindowArea (ha)— the spatial extent of the site, and the area over which residences have been observed, following conventions for site boundary

definition in use in each region. In many cases, TotalArea is a polygon enclosing buildings and infrastructure, but in smaller-scale societies it often includes the artifact scatter or local landform surrounding the built features as well. WindowArea may be an area of excavation or geophysical survey in cases where residential buildings are not visible on the modern ground surface, or it may include the area of surface survey or remote mapping (via unmanned aerial vehicle or lidar) when surface remains are apparent. There are cases where TotalArea is known but WindowArea is not, and vise versa. When WindowArea is recorded, all associated residences are from within this area.

WindowHH, Min/MaxHH, PopMethod—the number of residences that occur within the WindowArea, the estimated total number of residences at the site, and the method used to estimate the total residences. If some of the residences encountered within the window area were either not measured or not measurable, WindowHH will be greater than the number of measured residences. Methods used by local fieldworkers to estimate Min/MaxHH include total residence count, residential density extrapolated to the total site area, and other more complex methods.

LandLabor (Twoscale, Threescale, Fourscale)—the primary limiting factor (land or labor) for increasing production, expressed as a nominal variable and on 2-to-4-point ordinal scales. Contributors rated each site along these dimensions, following (3).

Delta dates (BCE/CE) *(Plant cultivation, Animal management, Portage, Riding, Traction, Enslavement, Copper smelting, Bronze, Writing, Massacres, Weights and measures, Marketplaces, Currency, Ostentatious individual burial, Intra-site public buildings, Specialized production of goods, Iron smelting, Complex bow)*—the date of earliest appearance and common occurrence of the listed attributes. This information was compiled by contributors at the Subregion level and is used to calculate the temporal span between these events and the residences at a given site.

Data collected for each residential building within each site include the following:

StructureID—the label given to a building in the source material.

HouseGroup—spatial division within which the structure occurs. In large, intensively investigated sites fieldworkers often subdivide the site area into smaller spatial units based on characteristics of the architecture, associated artifacts, built infrastructure, topography, or accessibility. These spatial units can be treated as neighborhoods or subdivisions of the site.

HouseType—a classification of building types: 1) Nuclear family; 2) Extended family; 3) Plazuela group (Maya); 4) Multi-family; 5) Men's house (New Guinea); 6) Women's house (New Guinea); 7) Storage Building; 8) Ceremonial building; 9)

Other; 10) Megastructure (E Europe); 11) Community Structure; 12) Surface Structure; 13) Pit Structure; 14) Apartment Compound (Central Mexico); 15) Public Building; 16) Palace; 17) Villa (Roman); 18) Agricultural Terrace; 19) Cuidadela (Central Mexico); 20) Cercadura (Central Andes); 21) Compound (Central Andes). Some of these are clearly not residential, whereas others combine residential and other administrative or ceremonial functions. For the analyses in this paper, we removed ceremonial and public buildings from consideration. This variable also seeks to capture variation in residential group composition by distinguishing nuclear family residences from extended and mult-family residences. Because it is not possible to determine the number of individuals who lived in a residential building consistently, the unit of population in the analyses of this paper is the residential building. It is rare for different types of household residences to occur within a single settlement, and residential building counts are generally proportional to settlement population within societies (4, 5). So, residential building count is a reasonable proxy for total population across settlements in a society.

HousePeriod, HousePhase—nested culture/historical groupings defined by research traditions in each region. In combination with geographical groupings at the site level, these fields provide a basis for aggregating residences by generally accepted geographic and temporal groups. In this paper, Region and HousePhase are generally used to create these groups.

BeginHouse, EndHouse (BCE/CE)—beginning and ending date of the buildings. BCE dates are negative. For some regions these dates duplicate the site-level dates, but in others these are distinct and generally more precise.

NRooms, NStories—when determined, the number of enclosed roofed spaces in the residence and the number of stories in the residence. Upper stories must be inferred from architectural traces in most cases, and we deferred to regional expertise for these inferences. Most residences in the database are single story, with multi-story residences being recorded only from a few regions. Multi-story dwellings are under-represented in the database due to preservation issues. For some Southern Andean sites, NRooms records the number of living rooms in the residence and NStories records the number of granaries. These cases are identified in the comments.

TotalAreaHouse, RoofedArea, UnroofedArea (m^2)—the total area encompassed by the residence, the unroofed area within the residence, and the area covered by roofs. All are totals at the residence level. Of the roughly fifty thousand residences in the database, roofed area is distinguished for about half, and unroofed area is noted for about twenty percent.

LivingArea (m^2), *AnimalArea* (m^2), *StorageArea* (m^2), *StorageVolume* (m^3)—the total area inferred to be living space, spaced devoted to keeping domestic animals, and space dedicated to storage, within the residence. Most multifunctional space was classified as living space. Intra-residence animal area

was often noted as definitely absent, and it is only recorded as definitely present in three sites. Storage area/volume refers to only to space that was specialized for, and dedicated to, storage. Storage rooms and granaries are inferred based on architectural information (restricted entry or accessibility, small size, absence of thermal features) or contents (storage vessels, internal storage features, traces of stored produce). For a few regions, storage facilities take the form of storage pits, or the height of storage structures can be determined. In these cases, the storage volume is also recorded. The only case where storage area/volume is recorded as zero is when storage area/volume is distinguished across the residences of a site and none is present in a specific residence. Compilers deferred to local expertise regarding the functional classification of spaces within residences in all cases. Of the roughly fifty thousand residences in the database, living area was distinguished for about 8500, and storage area/volume was distinguished for about 2900.

LaborMultiplier—this is a 3-point ordinal scale of construction quality intended to capture the relative labor investment in construction of a residence, per unit area. The rating is relative to the region and phase of residences, with a default value of one. This rating was recorded for approximately two-thirds of the residences in the database.

DistanceToCenter (m), *TypeOfCenter*—the straight-line distance from the residence to the central place in its settlement, and the type of feature used to represent the center. In some cases, the center is modeled as the geographic center of the settlement; in others it is a central plaza, a temple or palace, a crossroads, or a prominent civic-ceremonial building. Compilers followed regional expertise in determining what the center should be. These data are available for approximately twenty percent of residences.

MoundHgt (m)—the height of a residential mound relative to the surrounding terrain. This information was captured only for Maya settlements documented through lidar survey.

References—there are up to two references listed for each site, and for each residence within each settlement. References are more commonly recorded at the site level, and in some cases site and residence references are the same. Bibliographic information for all references cited in the database have been input into an open-access Zotero bibliography that can be accessed via: https://www.zotero.org/groups/5479570/gini_database/library.

Biases in the Database

The archaeological record is intrinsically biased due to several factors, from taphonomic processes affecting burial and decomposition to the accessibility of archaeological remains and investigator biases. In some parts of the world, such as the Southern Andes, ancient settlements are highly visible on the modern ground surface and can be reliably measured without excavation. In other parts,

such as Great Britain, settlements are concealed by ground cover or modern development, such that the remains can only be observed through geophysics or excavation. In some regions, like Central Mexico, modern agriculture has degraded architectural features even as they continually bring artifacts to the surface for inspection. In others, like the Maya region, dense jungle obscures artifacts but lidar is excellent for mapping architecture. Finally, in richer countries cultural heritage laws have spawned private industries that document archaeological sites for research and posterity, whereas in poorer countries only the most fabulous sites have been investigated, most often by foreign researchers.

In many parts of the world the sample of mapped and measured residential buildings is highly biased. Elite residences tend to be larger, more prominent, better preserved, and contain more spectacular art and finds. As a result, archaeologists have historically spent more time and effort documenting such buildings. The result is an oversampling of rare elite residences, and under sampling of much more common but less well-preserved commoner residences.

Two developments are ameliorating these biases to some extent. The first is the development of surface archaeology, through which archaeologists document all the buildings at a settlement through intensive surface mapping or remote sensing (lidar, aerial photography, or geophysical prospection). Such methods have been especially successful in the Southern Andes, the Maya region, the US Southwest, and Eastern Europe. The second is the development of cultural resource/heritage management in the United States and Canada, Great Britain, EU Nations, Korea and Japan. The laws and policies governing these industries mandate that archaeologists document all sites potentially impacted by new development and excavate sites that will be destroyed by such development. Because larger and more spectacular sites are more expensive to mitigate, developers increasingly steer clear of them. As a result, most excavations occur in relatively small sites that were ignored in the past, and the cumulative result is a much better representation of entire past societies.

Patterns of data availability relative to settlement size nevertheless remain complex. Ancient cities that have modern cities on top of them are often known only through numerous small excavations associated with new buildings, road construction, or utility work. In contrast, ancient cities that are no longer inhabited are increasingly being mapped in their entirety using various forms of remote sensing. Smaller sites, in contrast, often remain either entirely unknown or completely excavated, depending on the situation.

Although it is possible to accurately measure the dimensions of an individual residence, it is not possible to control for biases in the available samples of residences across sites, regions and time periods. Readers should therefore assume that every summary measure calculated for this paper contains an unknown and unquantified level of error. Nevertheless, these errors should be less biased when considered across all sites, regions, and time periods because

errors in different directions across cases should cancel each other out at this scale due to the law of large numbers. This is why the analyses in this paper, and others of the Special Feature, focus on generalization across cases as opposed to detailed analysis of specific cases.

Comments on Minimal Sample Sizes

Careful readers will notice that we utilize different minimal sample sizes in the various analyses conducted in this paper. These choices sought to balance group sizes against the estimates to be calculated. While it would be simpler to utilize a single minimal sample size, this would have been counterproductive due to patterns in missing data within the database. A summary of these choices and the reasoning behind them follows:

*House type, residence area and inequality (*Fig. S2)—To be included in this analysis the information from a given site needed to include at least five measured residential buildings, and at least two distinct values among these measurements, as this was deemed the minimal criteria for calculating distributional measures such as the Gini coefficient or standard deviation. These criteria are also reflected in the SiteGiniLevel.csv file available in the project archive.

Scaling of total residence area with residence count, by site (Fig. 2)—To be included in this analysis, the following information needed to be available: 1) at least one measured residence; and 2) either a) a total residence count or b) a combination of site area, window area, and window residence count. Because the goal is to estimate the elasticity of the relationship between residence count and total residence area by region, it was concluded that the analysis would be robust to errors in the mean residence area estimate for each site because some would be over-estimates while others would be under-estimates, and these errors would be independent of errors in the total residence count estimates. In addition to these criteria, various groups were removed from the analysis for a variety of reasons, as discussed in the Materials and Methods of the Main Text.

Baseline productivity, by region and period (Fig. 3)—Because this analysis carries forward results from the scaling analysis above, the same criteria for inclusion were used.

Distribution of residence areas, by region and period (Figs. S4, S5)—The scale of aggregation for this analysis is sufficient that no minimum sample size threshold is needed.

Covariance of current productivity and growth in productivity for inequality, by region and phase (Fig. 4, Fig. S6-8)—Deciles were used to assess changes in distributions of residence area across subsequent phases within each region. The minimum number of residences required to compute a mean residence size by decile is ten measured residences and at least two distinct values across

these ten measurements. Because these deciles are re-aggregated in the calculation of the covariance of residence area with growth in residence area, it was concluded that this minimal sample size would be adequate.

Residence area vs. distance to settlement center (Figs. 2A, S3)—In Figure S3a the linear model is calculated for all measured and plotted residences within each site. Approximately 30 observations are needed to satisfy the central limit theorem, making the confidence interval meaningful, so 30 is the minimal sample size in Figure S3a. In Figures 2A and S3b, the data are centered by site to control for variation in average residence areas and transportation modes across settlements. Because a single linear model is calculated for the entire dataset after centering by site, the analysis effectively weights the contribution of each site by sample size, making it feasible to include even very small samples. The minimum sample size required to calculate a slope is 2 observations, so this was chosen as the minimum sample size threshold.

Supplementary Figures

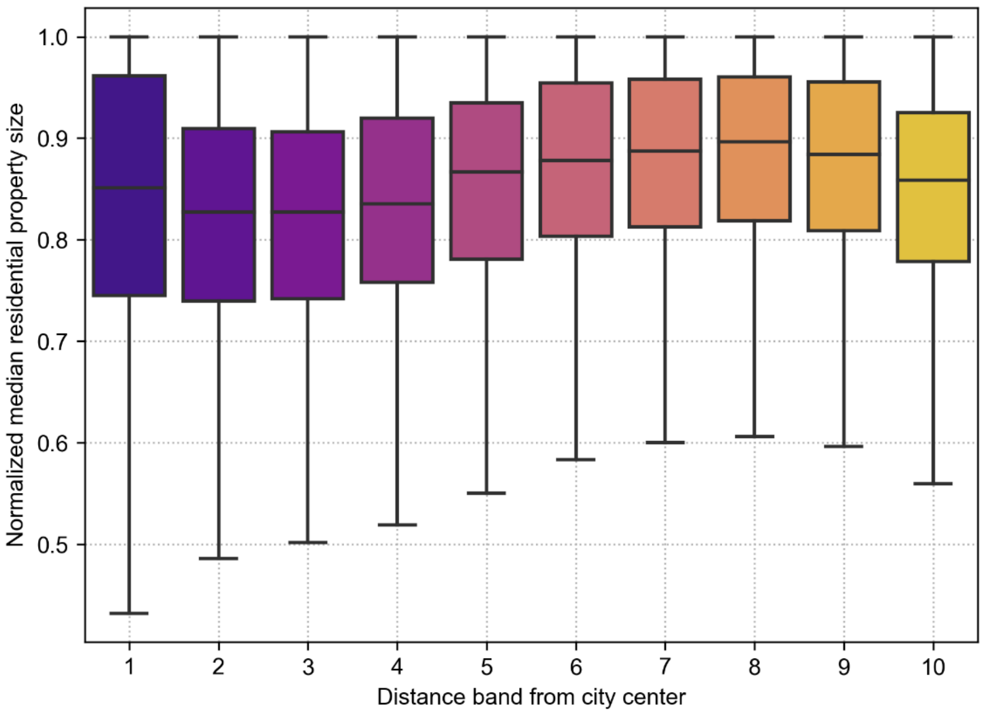

Fig. S1. Relationship between residence area and distance to the center, as observed in CBSAs in the continental United States in 2010. Distance band 1 is closest, and distance band 10 is farthest away from the barycenter of properties per CBSA. Distance bands are centered by CBSA. Using CBSA boundaries, and ZTRAX records of properties built by 2010, we identified, for each CBSA, single family residential property records from ZTRAX within the counties comprising the CBSA. We calculated the barycenter of properties using the centroid (i.e., mean x and y location of properties per CBSA) and computed the Euclidean distance of each property to the CBSA property barycenter. To account for the difference in absolute size of CBSAs, we then applied a decile-based ranking of properties for each CBSA. For each decile group, representing distance bands from the CBSA barycenter, in each CBSA, we then recorded the median indoor area of the properties within each distance band per CBSA. Finally, in order to account for absolute differences in property size between CBSAs, the median values of each CBSA are normalized into the range [0,1]. The boxplots in Fig. S1 show the distribution of normalized median property sizes per distance band across all CBSAs. Note the gradual increase in median residence size with distance, out to an inflection point in the periphery of the CBSA. Pairwise Kruskal-Wallis tests show that the differences in group medians are significant at the $P<.05$ level for the transition between distance bands 1-2, 3-4, 4-5, 5-6, and 9-10.

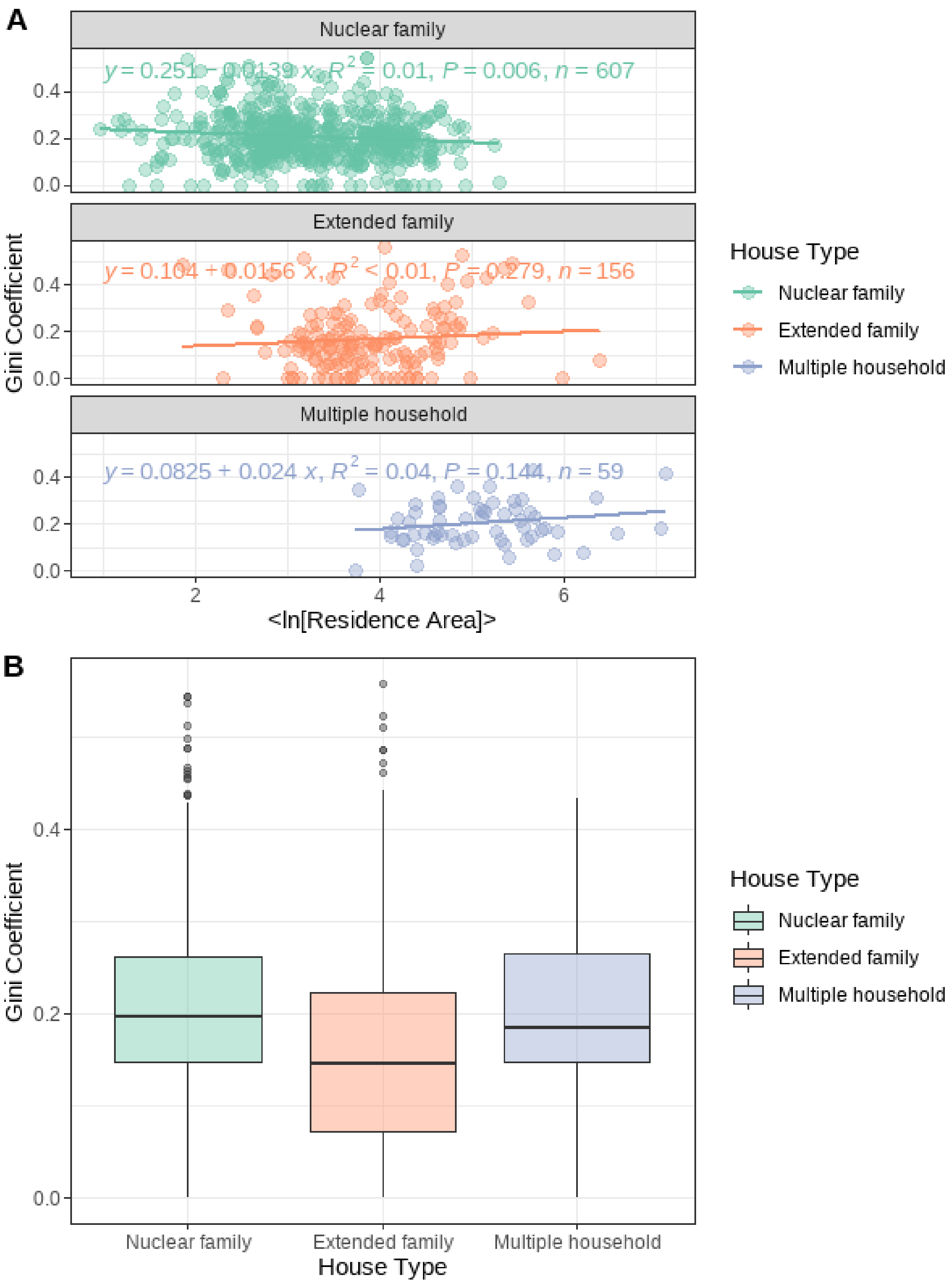

Figure S2. Relationship between residence type and size across settlements from small-scale (family-level, local, big-man, and simple chiefdom) societies in the GINI Project database. A) mean residence size vs. Gini coefficient across settlements with different characteristic residence types, note that extended and multiple family residences are generally larger, but the relationship between typical residence size and Gini coefficient is weak to nonexistent; B) Gini coefficients across settlements with different residence types, note that the distributions overlap substantially.

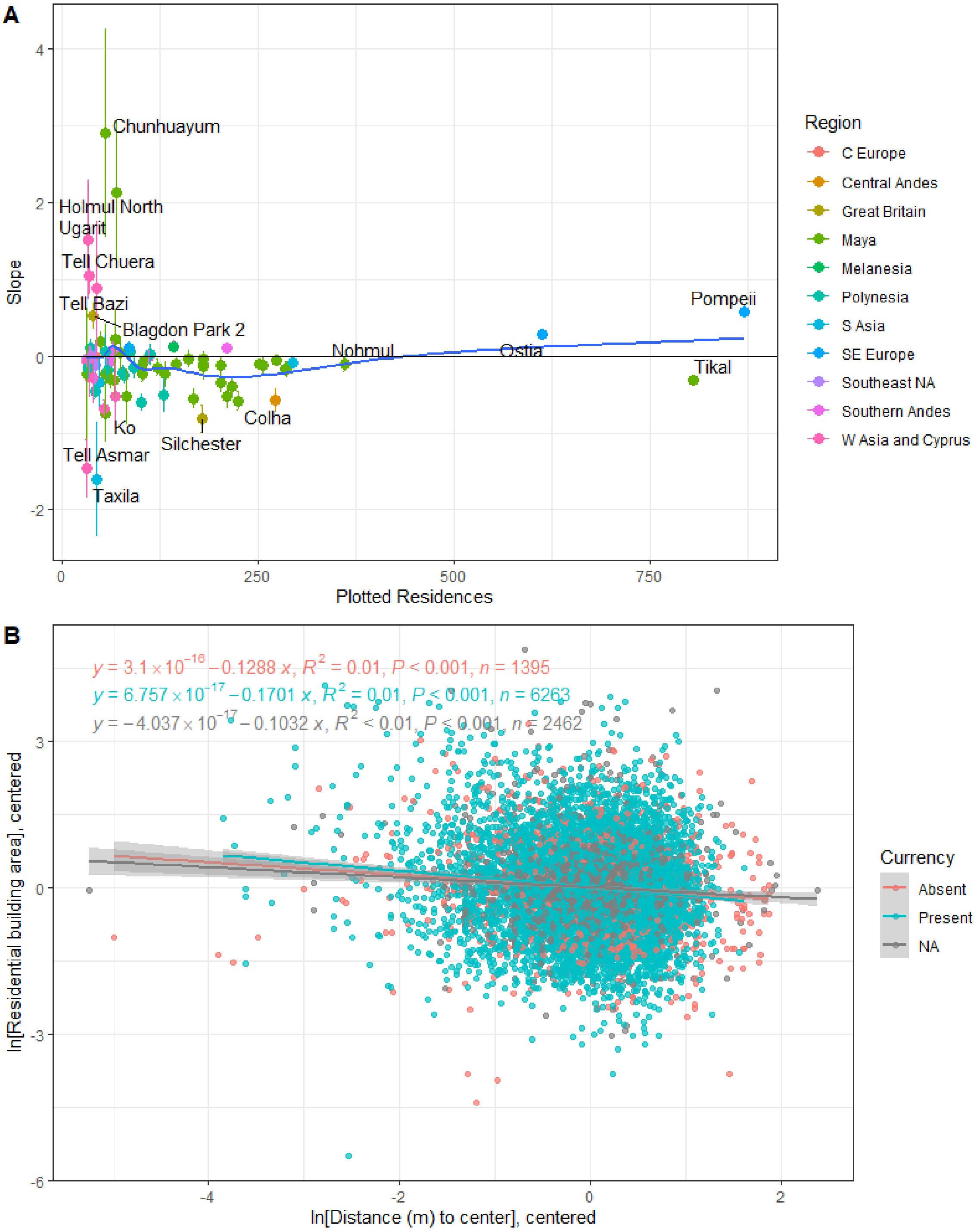

Fig. S3. Relationships between residence area vs. distance to the center; (A) slope (point estimate and standard errors) of the residence size - distance relationship vs. sample size for settlements with at least 30 plotted residences, with loess fit (span=.5), note that the average slope among settlements with precise estimates is negative, with the notable exception of Imperial Roman towns in Italy (Ostia, Pompeii); B) residence size vs. distance to center for all sites except Ostia and Pompeii, the gray bands represent one standard error of the slope estimate, note that the proxy for a monetized economy has no appreciable effect on the pattern.

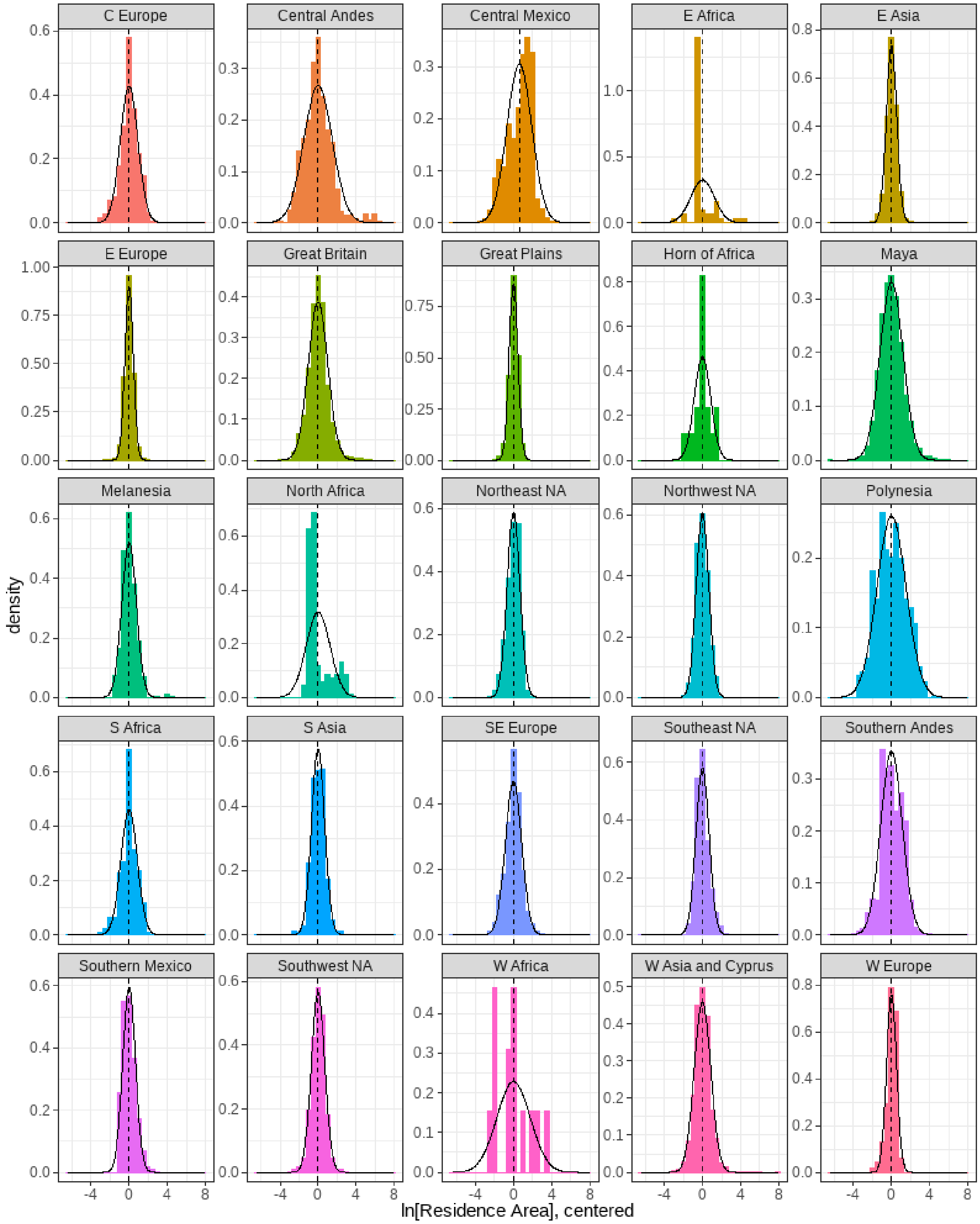

Fig. S4. Histograms of log-transformed residence areas, standardized by period within regions. The distributions for most well-sampled regions are approximately lognormal, as is typical of social quantities. Yautepec is excluded from Central Mexico because in the source data 1,596 of 1,619 houses have the same area.

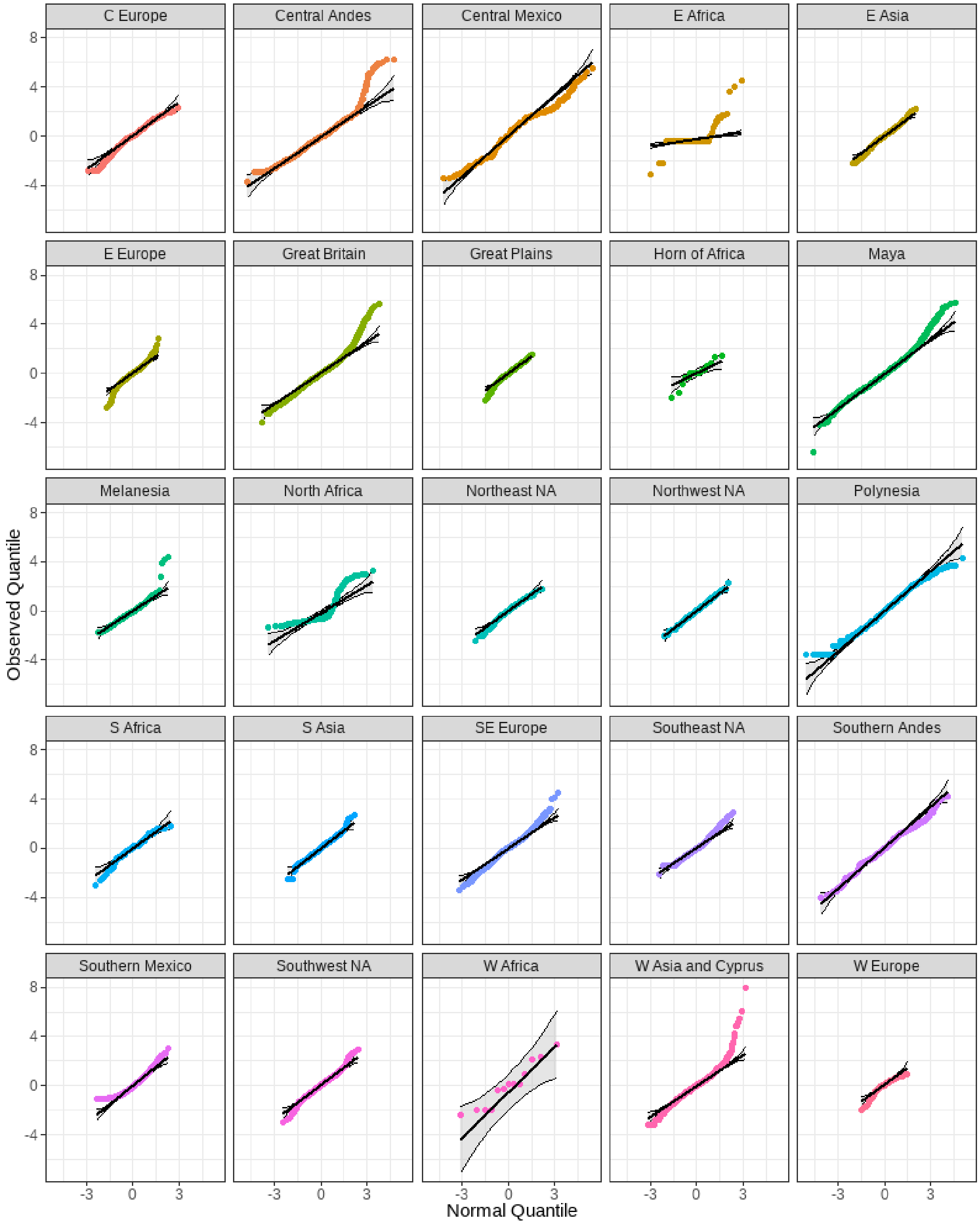

Fig. S5. QQ-Plots of log-transformed residence areas, standardized by period within regions. The tails for several regions extend beyond the 95% confidence bands for a normal distribution, shown in gray. We interpret this as evidence of over/undersampling of the tails of the residence size distribution by archaeologists.

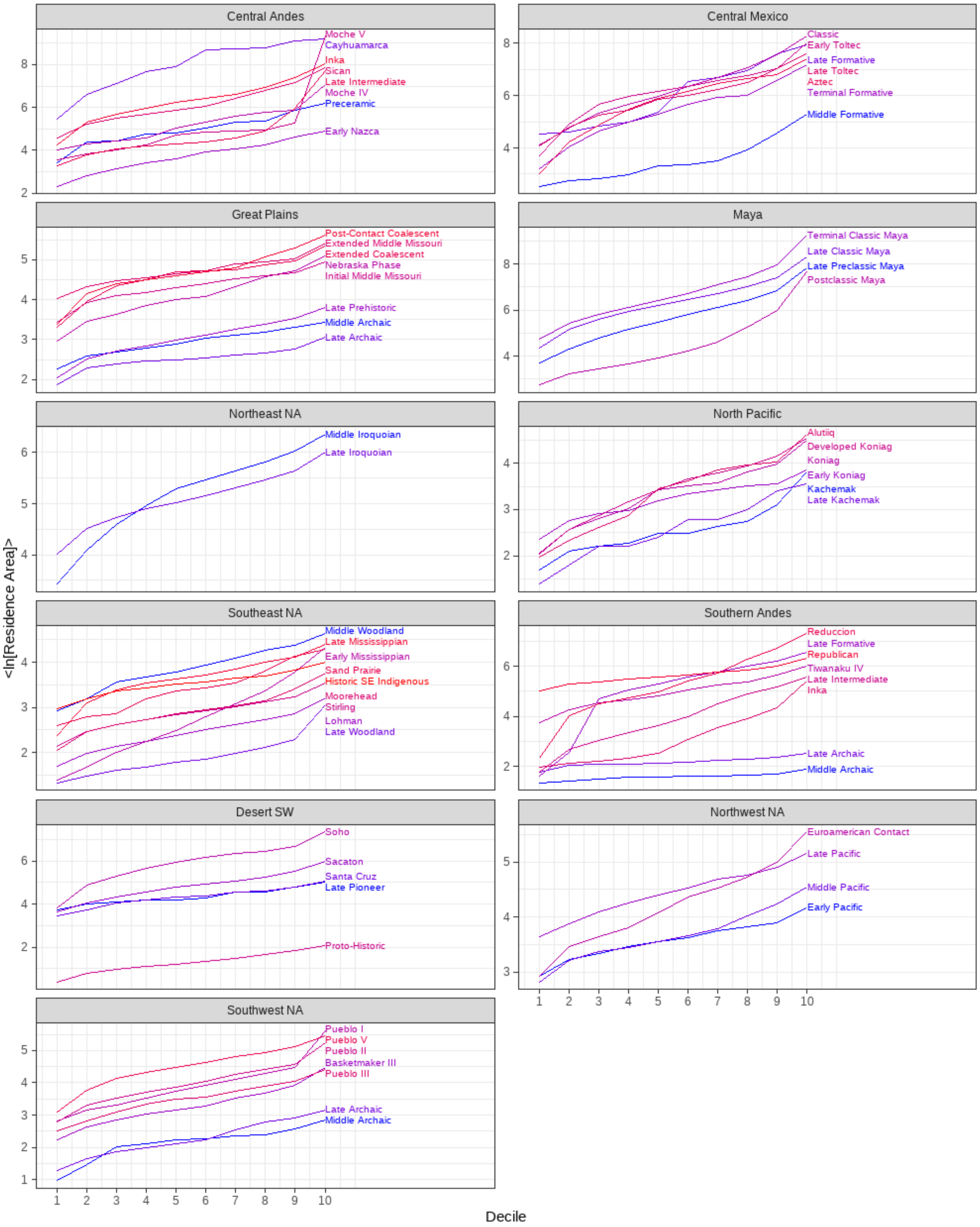

Fig. S6a. Cumulative distributions of residence areas, by region phase, and decile, for western hemisphere subregions. Colors associated with lines and labels correspond to the order of the regional sequence.

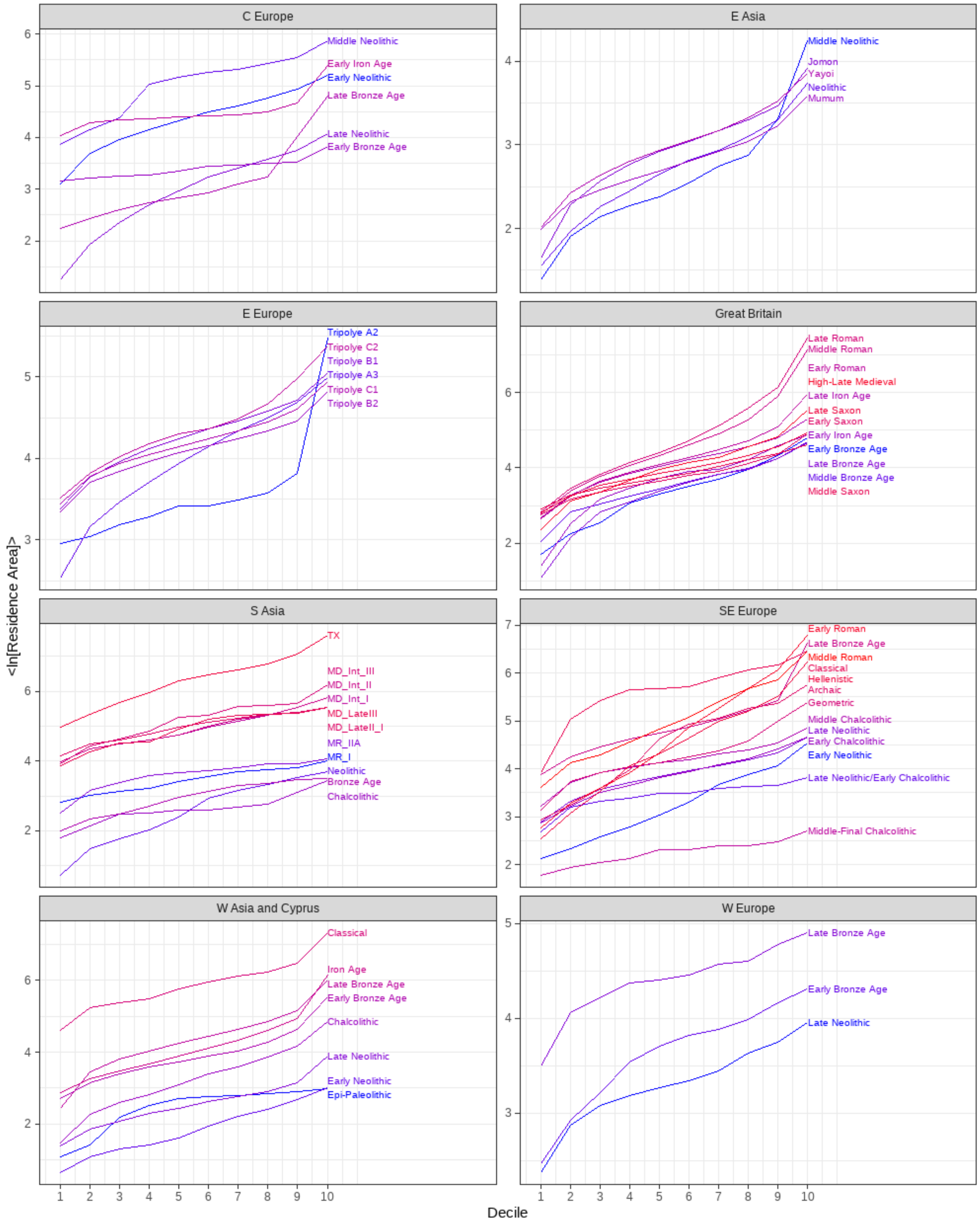

Fig. S6b. Cumulative distributions of residence areas, by region phase, and decile, for eastern hemisphere subregions. Colors associated with lines and labels correspond to the order of the regional sequence.

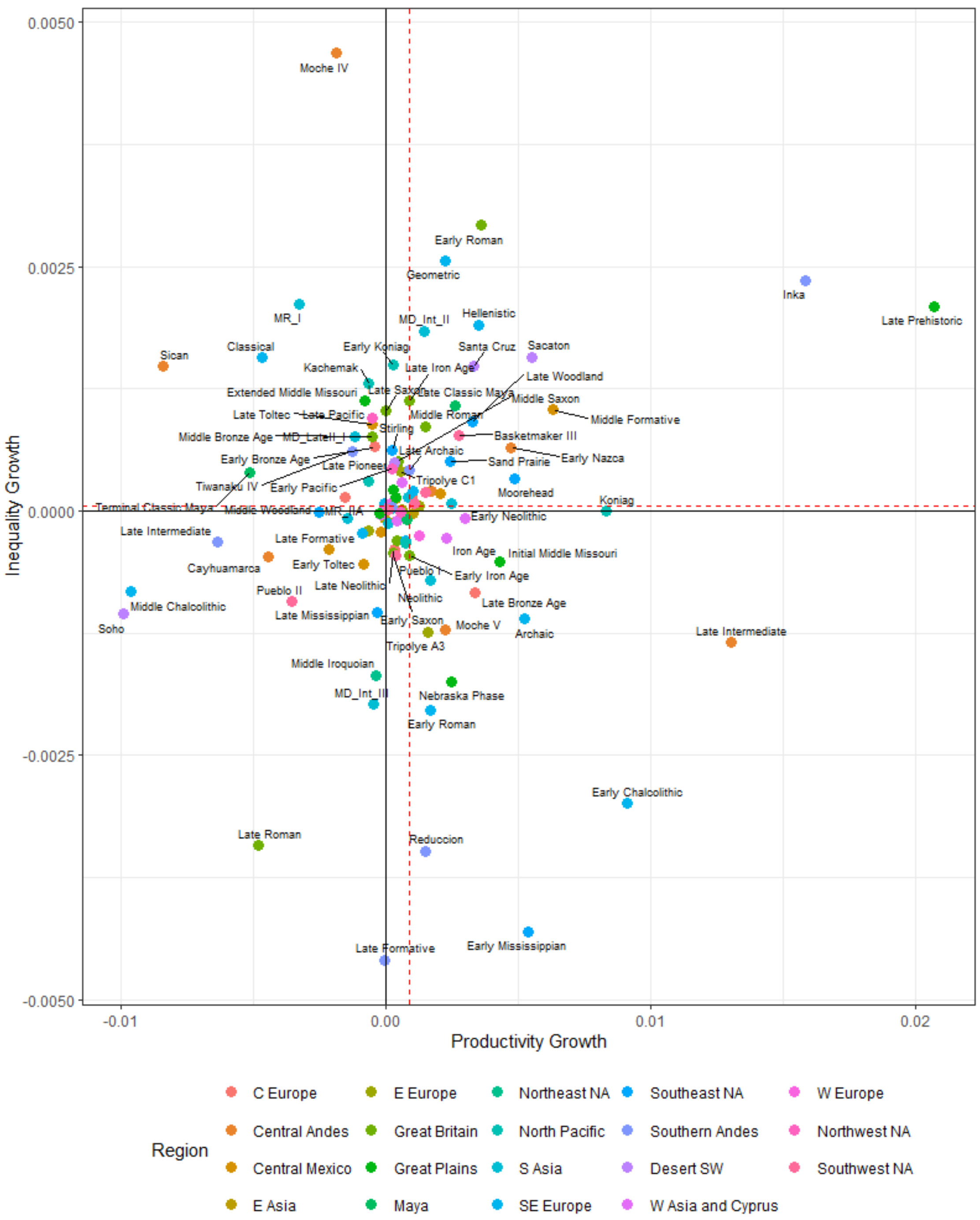

Fig. S7. Relationship between productivity growth and inequality growth across regions and phases. Growth rates are from the labeled phase to the subsequent phase in the regional sequence, with the latter indicated by symbol color. The dashed red lines show the mean coordinate of each measure. All four quadrants are filled, indicating that productivity growth and inequality growth are uncorrelated across societies. Also note that mean productivity growth (change in mean-log residence size) across all transitions is slightly positive, whereas mean inequality growth (change in standard deviation-log of residence size) is essentially zero. This pattern suggests net increases in productivity (environmental learning, broadly construed), but no net trends in inequality characterize human history overall.

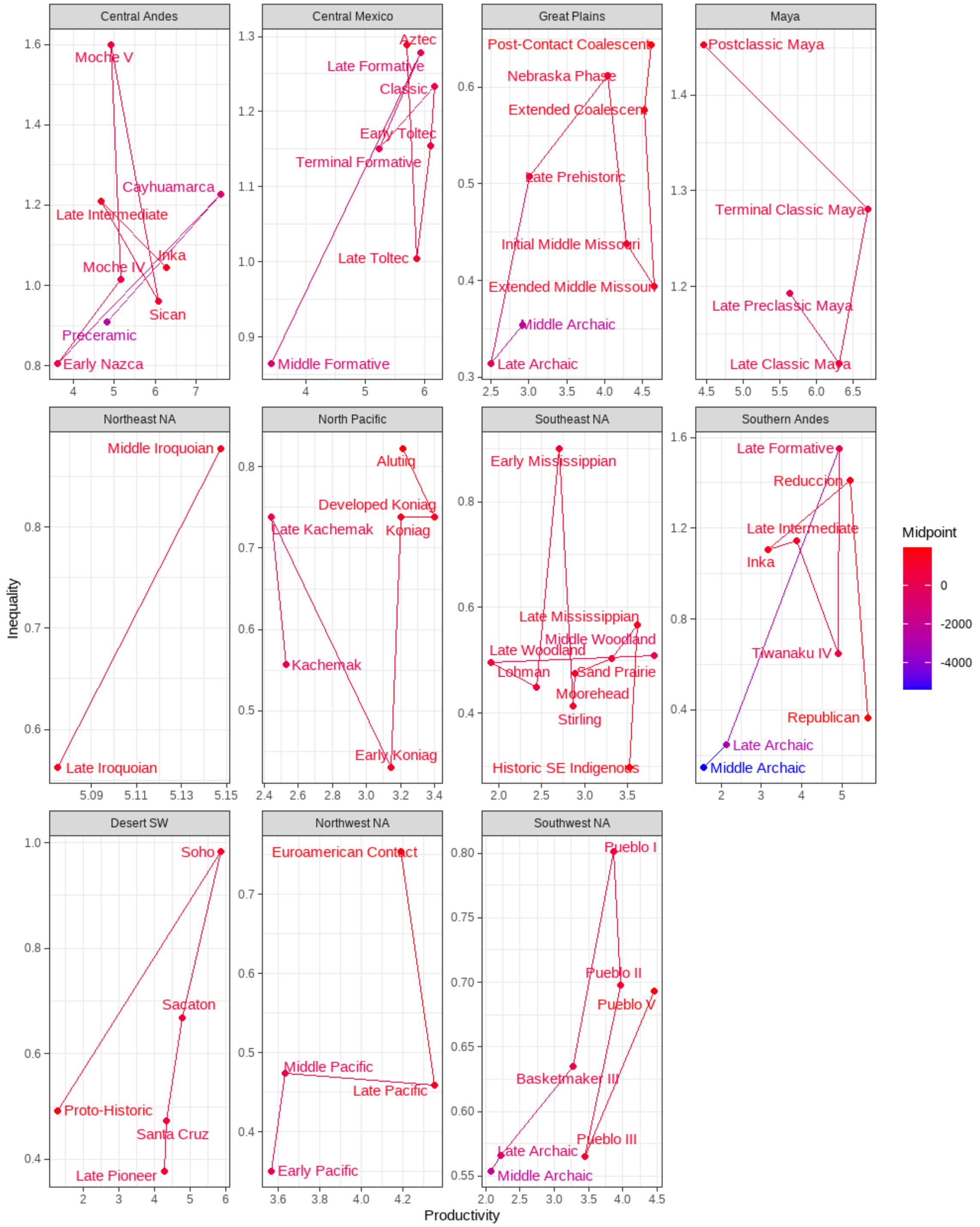

Fig. S8a. Phase plot of productivity (mean of the log of residential building area) vs. inequality (standard deviation of the log of residential building area) by phase, for western hemisphere subregions. Colors are indexed to the midpoint of the occupations of sites assigned to each phase.

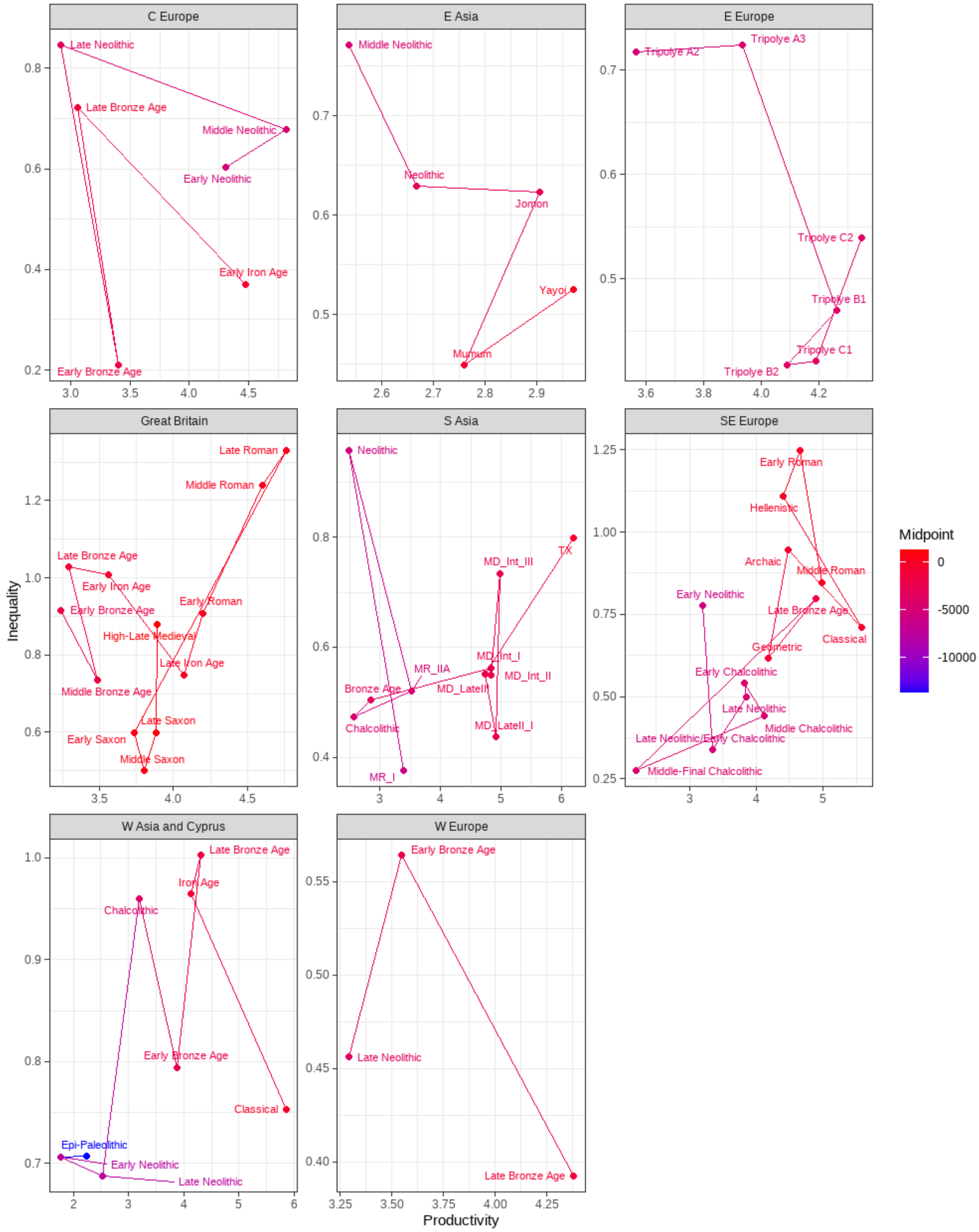

Fig. S8b. Phase plot of productivity (mean of the log of residential building area) vs. inequality (standard deviation of the log of residential building area) by phase, for eastern hemisphere subregions. Colors are indexed to the midpoint of the occupations of sites assigned to each phase.

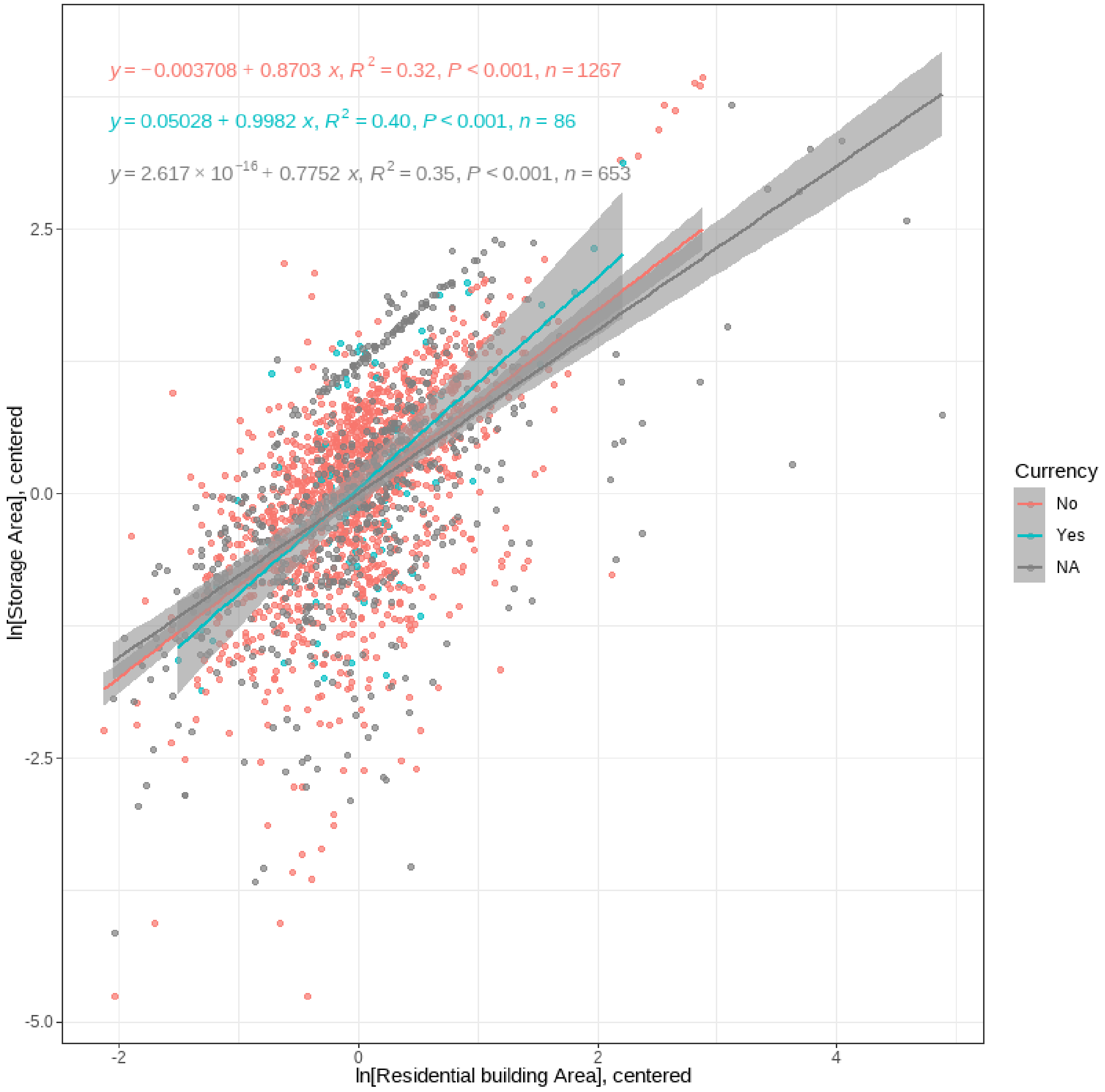

Fig. S9. Storage area vs. total area for individual residences where storage space is distinguished in the source data. The gray bands represent one standard error in the slope estimate. Cases are grouped according to the presence vs. absence of currency in the associated society, as determined by regional experts. The slope for residences associated with currency is close to one, but the standard error is large and overlaps with those of the other groups. The analysis thus does not provide statistical evidence that the emergence of a monetized economy affects the relationship between residence area and storage area.

Supplementary Tables

Table S1. Scaling of total residence count with total residence area, by region.

**Region**	***Beta***	***SE***	***Intercept***	***SE***	R^2	***F***	***P***	***N***
Andes	1.168	0.121	3.448	0.624	.680	93.57	1.84E-12	46
S Africa	1.215	0.335	2.241	1.195	.725	13.17	0.015066	7
W Asia and Cyprus	1.165	0.068	2.763	0.361	.776	294.77	2.29E-29	87
C Europe	1.122	0.142	3.653	0.592	.827	62.15	2.62E-06	15
Central Mexico	1.244	0.116	4.542	0.528	.810	115.24	3.03E-11	29
E Asia	0.976	0.059	2.798	0.404	.959	277.20	1.17E-09	14
E Europe	1.071	0.031	3.724	0.174	.995	1231.05	3.57E-08	8
Great Britain	1.243	0.078	3.321	0.451	.908	256.67	5.47E-15	28
Great Plains	1.452	0.165	2.632	0.657	.886	77.49	5.05E-06	12
Maya	1.014	0.140	6.135	0.707	.572	52.19	1.06E-08	41
Northeast NA	0.893	0.146	5.354	0.389	.531	37.32	7.00E-07	35
Northwest NA	1.029	0.174	3.568	0.430	.416	34.92	3.23E-07	51
SE Europe	1.230	0.092	3.073	0.508	.877	178.90	6.78E-13	27
Southeast NA	0.999	0.078	3.549	0.323	.864	164.54	9.43E-13	28
Southern Mexico	1.773	0.375	-1.409	2.770	.957	22.34	0.132747	3
Southwest NA	1.130	0.146	3.818	0.541	.770	60.31	3.73E-07	20
Total (Centered)	1.156	0.029	-3.84E-17	4.15E-02	.781	1601.00	<2.2E-16	451

Table S2. Scaling of storage area with total residential building area for individual residences, by region.

**Region**	***Beta***	***SE***	***Intercept***	***SE***	R^2	***F***	***P***	***N***
C Europe	1.172	0.107	-2.018	0.483	.587	120.86	5.26E-18	87
E Asia	0.228	0.230	0.793	0.646	.022	0.98	3.27E-01	46
Great Plains	0.740	0.279	-2.785	1.316	.115	7.02	1.06E-02	56
Melanesia	1.184	0.522	-1.468	2.109	.631	5.14	1.08E-01	5
Northeast NA	0.667	0.051	0.466	0.278	.477	173.89	1.18E-28	193
Northwest NA	1.195	0.077	-2.048	0.279	.668	241.86	1.53E-30	122
SE Europe	0.787	0.142	-0.599	0.875	.754	30.71	2.47E-04	12
Southern Andes	0.672	0.049	-1.417	0.251	.201	190.51	7.92E-39	761
Southwest NA	1.291	0.059	-2.255	0.218	.583	484.08	1.00E-67	348
W Asia and Cyprus	0.706	0.053	-0.545	0.243	.319	174.93	4.99E-33	376
Total (centered)	0.829	0.025	-5.87e-17	1.861e-02	.339	1026	<2.2e-16	2006

Table S3. Sites for which distances to central places have been recorded.

Site	Begin Date	End Date	Region	Subregion	Measured Houses	Median Size (m2)	Median Distance (m)	Central Place
Mehrgarh	-6500	-4700	S Asia	Indus River Basin	47	35	12	Geometrical Centre of Mound
Mohenjo-daro	-2500	-1900	S Asia	Indus River Basin	26	225	165	Geometrical Centre of Citadel Mound
Mohenjo-daro	-2500	-1900	S Asia	Indus River Basin	188	127	240	Geometrical Centre of Northern Mound
Mohenjo-daro	-2500	-1900	S Asia	Indus River Basin	80	142	75	Geometrical Centre of Southern Mound
Taxila	-600	-200	S Asia	Indus River Basin	14	312	150	Geometrical Centre of Dargah Mound
Taxila	-200	-100	S Asia	Indus River Basin	31	730	150	Geometrical Centre of Monastery Mound
Çayönü	-7500	-7200	W Asia and Cyprus	Anatolia	29	20	64	Plaza: monumental communal open space, with evidence of cultic activities + last stage of temple building (called 'terrazzo building') on the east side of the excavation area
Çayönü	-7900	-7600	W Asia and Cyprus	Anatolia	9	30	39	Temple BM2a
Çayönü	-8400	-8000	W Asia and Cyprus	Anatolia	15	46	31	Temple FA

Site	Begin Date	End Date	Region	Subregion	Measured Houses	Median Size (m2)	Median Distance (m)	Central Place
A??kl? Höyük	-8000	-7500	W Asia and Cyprus	Anatolia	42	10	44	Complex HV (During 2006, 19, 102)
Zincirli Höyük	-800	-750	W Asia and Cyprus	Anatolia	12	57	332	Citadel
Yanik Tepe	-2900	-2200	W Asia and Cyprus	Iran	11	14	9	Circle 1
Arad	-3000	-2650	W Asia and Cyprus	Levant	35	55	75	Sacred Precint in Area T
Beer-Sheba	-900	-701	W Asia and Cyprus	Levant	142	36	36	The City Square which is surrounded by the "Governor's House", the gate and the storage buildings.
Beidha	-7130	-6440	W Asia and Cyprus	Levant	14	78	22	Building 8 in Subphase C2
Khirbet al-Lahun	-1200	-1000	W Asia and Cyprus	Levant	15	31	74	Fortress
Shk?rat Msaied	-8250	-7950	W Asia and Cyprus	Levant	7	15	9	Unit F
Tell Beit Mirsim	-800	-700	W Asia and Cyprus	Levant	32	102	175	The Western Tower
Tell Masos	-1200	-1000	W Asia and Cyprus	Levant	13	73	47	Temple located on the northern part of the tell
Tell Mastuma	-900	-730	W Asia and Cyprus	Levant	39	30	31	Temple located at Block 4
Tell Qasile	-1051	-938	W Asia and Cyprus	Levant	8	73	56	Temple 131 in Area C
Ugarit	-1230	-1180	W Asia and Cyprus	Levant	33	194	251	The Acropolis
Wadi Faynan 16	-10600	-8200	W Asia and Cyprus	Levant	11	8	13	Structure O75 appears to be a community building (not included in this spreadsheet)

Site	Begin Date	End Date	Region	Subregion	Measured Houses	Median Size (m2)	Median Distance (m)	Central Place
Assur	-900	-614	W Asia and Cyprus	Mesopotamia	27	60	596	The Assur Tempel
Habuba Kebira South	-3300	-3100	W Asia and Cyprus	Mesopotamia	35	43	157	Temple Complex on Tell Qannas
Haradum	-1749	-1627	W Asia and Cyprus	Mesopotamia	16	44	24	La Place Centrale
Nuzi	-1500	-1400	W Asia and Cyprus	Mesopotamia	13	97	113	Central Temple
Tell Asmar	-2350	-2250	W Asia and Cyprus	Mesopotamia	22	43	121	Abu Temple
Tell Bazi	-1450	-1350	W Asia and Cyprus	Mesopotamia	44	155	280	The temple on the top of the Citadel
Tell Chuera	-2562	-2465	W Asia and Cyprus	Mesopotamia	22	29	78	2 different Centers = 1 -Temple at the centre of the site (Steinbau VI), 2 - The so called Temenos Area at Area A featuring two large temples built out of stone and a monumental staircase (Steinbau I & Steinbau II).
Tell Chuera	-2465	-2350	W Asia and Cyprus	Mesopotamia	13	99	228	3 different Centers = 1 - Palace at Area A, 2 - Temple at the centre of the site (Steinbau VI), 3 - The so called Temenos Area at Area A featuring two large temples built out of stone and a

Site	Begin Date	End Date	Region	Subregion	Measured Houses	Median Size (m2)	Median Distance (m)	Central Place
								monumental staircase (Steinbau I & Steinbau II).
Tell Halawa A	-2450	-2100	W Asia and Cyprus	Mesopotamia	14	79	75	Religious quarter on the south-east (Arcane Volume, 50)
Tell Munbaqa	-1600	-1200	W Asia and Cyprus	Mesopotamia	11	80	235	Temple Area located in the NW of the settlement
Tell Sabi Abyad	-1195	-1183	W Asia and Cyprus	Mesopotamia	19	30	24	"The Central Building"
Tell al-Raq?'i	-2750	-2600	W Asia and Cyprus	Mesopotamia	14	16	22	Round Building
Abu Salabikh	-2900	-2700	W Asia and Cyprus	Mesopotamia	12	272	517	Partially excavated palace on the South Mound
Babylon	-625	-539	W Asia and Cyprus	Mesopotamia	19	338	106	Ishtar Temple
Tell Asmar	-2250	-2150	W Asia and Cyprus	Mesopotamia	10	130	91	Abu Temple
Tell Harmal	-1900	-1750	W Asia and Cyprus	Mesopotamia	13	52	77	The quarter near the gate, which contains the temple and the administrative building (Verwaltungsgebaude)
Ur	-1850	-539	W Asia and Cyprus	Mesopotamia	68	83	366	Temenos Area
Schwanfeld	-5500	-5350	C Europe	Germany	7	143	37	central burial
Inden 1	-4650	-4550	C Europe	Germany	12	120	100	village plaza
Biskupin	-700	-400	C Europe	Poland	43	80	99	resource
Bath	60	400	Great Britain	Central Belt	2	206	77	Forum or crossroads

Site	Begin Date	End Date	Region	Subregion	Measured Houses	Median Size (m2)	Median Distance (m)	Central Place
Totternhoe	250	400	Great Britain	Central Belt	1	4964	3088	Forum or crossroads
Bancroft villa	100	450	Great Britain	Central Belt	6	142	265	Forum or crossroads
Broughton Manor Farm	-100	400	Great Britain	Central Belt	2	80	53	Forum or crossroads
Grendon Underwood (Plot 5.03-05)	1	400	Great Britain	Central Belt	3	79	1200	Forum or crossroads
Yewden Roman villa	-50	400	Great Britain	Central Belt	4	376	32	Forum or crossroads
Camp Ground	-100	400	Great Britain	Central Belt	22	75	95	Forum or crossroads
Godmanchester	80	400	Great Britain	Central Belt	1	60	1166	Forum or crossroads
Water Newton	120	400	Great Britain	Central Belt	1	181	1000	Forum or crossroads
Cirencester	160	410	Great Britain	Central Belt	20	185	190	Forum or crossroads
Claydon Pike	1	400	Great Britain	Central Belt	1	335	71	Forum or crossroads
Gloucester Business Park Link Road, Hucclecote	50	325	Great Britain	Central Belt	1	113	5500	Forum or crossroads
Kingshill North, Cirencester	-100	100	Great Britain	Central Belt	1	79	2300	Forum or crossroads
Portway	100	300	Great Britain	Central Belt	1	103	4000	Forum or crossroads
The Ditches	1	200	Great Britain	Central Belt	1	400	8000	Forum or crossroads
Elms Farm	-100	50	Great Britain	Central Belt	1	209	5000	Forum or crossroads
Cosgrove	100	400	Great Britain	Central Belt	2	250	51	Forum or crossroads
Glapthorn Road, Oundle	1	375	Great Britain	Central Belt	2	67	1200	Forum or crossroads
Higham Ferrers OA	100	400	Great Britain	Central Belt	10	78	67	Forum or crossroads
Irchester	50	300	Great Britain	Central Belt	3	12	361	Forum or crossroads

Site	Begin Date	End Date	Region	Subregion	Measured Houses	Median Size (m2)	Median Distance (m)	Central Place
Potterspury	-50	350	Great Britain	Central Belt	2	47	8000	Forum or crossroads
Alchester	45	500	Great Britain	Central Belt	7	76	962	Forum or crossroads
Dorchester-on-Thames	60	500	Great Britain	Central Belt	1	78	65	Forum or crossroads
Bradley Hill, Somerton	-100	450	Great Britain	Central Belt	2	107	8800	Forum or crossroads
Chesterton-on-Fosse	150	400	Great Britain	Central Belt	1	95	95	Forum or crossroads
Durocornovium	60	400	Great Britain	Central Belt	4	175	50	Forum or crossroads
Nettleton	50	400	Great Britain	Central Belt	7	200	83	Forum or crossroads
Worcester	80	350	Great Britain	Central Belt	1	42	356	Forum or crossroads
Wroxeter	80	400	Great Britain	Central West	14	285	75	Forum or crossroads
Alcester	50	400	Great Britain	Central West	17	72	450	Forum or crossroads
Braughing	-15	400	Great Britain	East	10	28	700	Forum or crossroads
Brampton	100	400	Great Britain	East	2	50	74	Forum or crossroads
Caistor St Edmund villa	100	450	Great Britain	East	1	373	47	Forum or crossroads
Carlisle	72	400	Great Britain	North	2	28	458	Forum or crossroads
Blagdon Park 2	-100	100	Great Britain	North	40	39	81	Forum or crossroads
Woolaw, Upper Redesdale	100	200	Great Britain	North	4	42	1650	Forum or crossroads
Welton villa	-50	410	Great Britain	North-East	4	112	262	Forum or crossroads
Catterick	80	400	Great Britain	North-East	25	75	40	Forum or crossroads
Holmfield Interchange, A1(M), Ferrybridge (incl. Site P)	-50	350	Great Britain	North-East	5	48	148	Forum or crossroads

Site	Begin Date	End Date	Region	Subregion	Measured Houses	Median Size (m2)	Median Distance (m)	Central Place
Alington Avenue, Fordington	1	400	Great Britain	South	2	71	1000	Forum or crossroads
Chelmsford	50	400	Great Britain	South	3	65	192	Forum or crossroads
Bitterne	170	390	Great Britain	South	1	39	496	Forum or crossroads
Dunkirt Barn	-50	400	Great Britain	South	5	494	36	Forum or crossroads
Neatham	75	400	Great Britain	South	4	39	30	Forum or crossroads
Silchester	50	450	Great Britain	South	178	260	235	Forum or crossroads
Silchester, Insula IX	50	450	Great Britain	South	16	94	160	Forum or crossroads
St Albans Early	60	160	Great Britain	South	19	155	135	Forum or crossroads
St Albans Initial	43	60	Great Britain	South	1	208	287	Forum or crossroads
St Albans Late	275	450	Great Britain	South	13	180	121	Forum or crossroads
Darenth	125	375	Great Britain	South	2	7689	73	Forum or crossroads
Lullingstone	100	420	Great Britain	South	1	510	17	Forum or crossroads
Rochester, Havisham Centre	80	220	Great Britain	South	1	24	300	Forum or crossroads
Rapsley	80	350	Great Britain	South	5	372	20	Forum or crossroads
Reawla, Gwinear	100	325	Great Britain	South-West	1	133	800	Forum or crossroads
Exeter	80	400	Great Britain	South-West	18	77	168	Forum or crossroads
Zagora	-900	-700	SE Europe	Greece	14	85	39	Temple
Athens	-500	-400	SE Europe	Greece	7	149	175	Agora
Dema	-420	-400	SE Europe	Greece	1	474	11450	Agora
Halai Axonides	-500	-300	SE Europe	Greece	6	143	405	Agora
Lathouriza	-700	-500	SE Europe	Greece	8	37	18	Sanctuary
Thorikos	-700	-300	SE Europe	Greece	6	285	100	Theater
Vari	-325	-275	SE Europe	Greece	1	255	15280	Agora
Olynthos	-432	-316	SE Europe	Greece	85	327	172	Agora

Site	Begin Date	End Date	Region	Subregion	Measured Houses	Median Size (m2)	Median Distance (m)	Central Place
Emporio	-700	-600	SE Europe	Greece	10	30	144	Athena Temple
Anavlochos	-1000	-700	SE Europe	Greece	2	150	259	Sanctuary
Azoria	-600	-475	SE Europe	Greece	3	270	55	Monumental Civic Building
Kavousi Kastro	-1200	-600	SE Europe	Greece	18	48	33	Building H - elite dwelling/political or religious center
Lato	-700	-150	SE Europe	Greece	8	95	50	Agora
Trypetos	-250	-150	SE Europe	Greece	3	66	26	Prytaneion
Elea	-400	-167	SE Europe	Greece	7	160	60	Agora
Kassope	-350	0	SE Europe	Greece	2	243	90	Agora
Eretria	-800	-100	SE Europe	Greece	5	632	610	Agora
Vroulia	-650	-550	SE Europe	Greece	15	191	108	Agora (?)/plaza
Glykadi	-350	-300	SE Europe	Greece	1	650	5610	Agora
Limenas Thasos	-700	-200	SE Europe	Greece	4	255	590	Agora
Marmaromandra	-350	-300	SE Europe	Greece	1	320	2280	Agora
Pacbitun	600	900	Maya	Eastern Lowlands	162	535	506	Civic Ceremonial Core
Altun Ha	400	900	Maya	Eastern Lowlands	168	693	503	Civic Ceremonial Core
Aventura	750	1100	Maya	Eastern Lowlands	66	516	505	Civic Ceremonial Core
Colha	600	800	Maya	Eastern Lowlands	224	388	1003	Civic Ceremonial Core
Kohunlich	600	900	Maya	Eastern Lowlands	217	484	786	Civic Ceremonial Core
Nohmul	700	1000	Maya	Eastern Lowlands	360	688	1462	Civic Ceremonial Core
Saturday Creek	600	900	Maya	Eastern Lowlands	131	654	1069	Civic Ceremonial Core
Cauinal	1300	1600	Maya	Highlands	287	30	145	Civic Ceremonial Core
Zacualpa	1250	1530	Maya	Highlands	104	294	374	Civic Ceremonial Core

Site	Begin Date	End Date	Region	Subregion	Measured Houses	Median Size (m2)	Median Distance (m)	Central Place
Chunhuayum	-300	1000	Maya	Northern Lowlands	55	1505	3827	Civic Ceremonial Core Uci
Xtobo	-400	-250	Maya	Northern Lowlands	211	162	304	Civic Ceremonial Core
Sayil	800	1000	Maya	Northern Lowlands	285	449	712	Civic Ceremonial Core
Xkipche	650	770	Maya	Northern Lowlands	62	670	198	Civic Ceremonial Core
La Nueva	600	900	Maya	Pacific Coast	74	452	294	Civic Ceremonial Core
Nakbe	600	850	Maya	Peten	181	562	823	Civic Ceremonial Core
Tikal	600	900	Maya	Peten	805	657	1374	Civic Ceremonial Core
Topoxte	1100	1450	Maya	Peten	273	53	421	Civic Ceremonial Core Topoxte Island
Yaxha	600	900	Maya	Peten	251	667	3075	Civic Ceremonial Core
Zacpeten	1400	1700	Maya	Peten	146	58	142	Civic Ceremonial Core
Cival	-300	250	Maya	Peten	180	385	888	Civic Ceremonial Core
Dos Aguadas	-300	300	Maya	Peten	102	363	2147	Civic Ceremonial Core
Hamontun	600	900	Maya	Peten	202	297	1075	Civic Ceremonial Core
Holmul	600	900	Maya	Peten	256	258	1360	Civic Ceremonial Core
Holmul North	600	900	Maya	Peten	70	357	9871	Civic Ceremonial Core
Ko	600	900	Maya	Peten	56	246	281	Civic Ceremonial Core
Ix Kuku'il	-50	900	Maya	Southern Belize	121	602	1635	Civic Ceremonial Core
Lubaantun	700	900	Maya	Southern Belize	49	1007	281	Civic Ceremonial Core
Pusilha	571	900	Maya	Southern Belize	202	428	694	Civic Ceremonial Core
Uxbenka	-250	1000	Maya	Southern Belize	180	637	1864	Civic Ceremonial Core
Altar de Sacrificios	600	950	Maya	Western Lowlands	101	534	761	Main Plaza Group A
El Caoba	600	900	Maya	Western Lowlands	31	348	7477	Main Plaza Group A
El Pabellon	600	900	Maya	Western Lowlands	55	610	4014	Main Plaza Group A
El Trapiche	600	950	Maya	Western Lowlands	68	689	2559	Main Plaza Group A

Site	Begin Date	End Date	Region	Subregion	Measured Houses	Median Size (m2)	Median Distance (m)	Central Place
Ixcoche	600	950	Maya	Western Lowlands	82	801	4478	Main Plaza Group A
Los Puerquitos	600	900	Maya	Western Lowlands	11	416	3244	Main Plaza Group A
Vega de la Pista	600	900	Maya	Western Lowlands	26	1079	10309	Main Plaza Group A
Alderson Farm	1420	1500	Northeast NA	Neutral	4	200	50	centerpoint
Brasstown Valley	-150	1600	Southeast NA	Deep South	32	29	233	Geographic
Coweeta Creek	1300	1600	Southeast NA	Deep South	12	48	36	Mound
Toqua	1200	1800	Southeast NA	Deep South	36	37	101	Mound A
Townsend	1625	1800	Southeast NA	Deep South	9	37	235	Geographic
Warren Wilson	1250	1450	Southeast NA	Deep South	12	40	10	Plaza
Duncan Tract	-200	200	Southeast NA	Deep South	5	95	33	Geographic center
Jewell	1000	1200	Southeast NA	Deep South	11	64	21	Mound
Morris	1000	1300	Southeast NA	Deep South	12	43	26	Plaza
Yearwood MW	-200	500	Southeast NA	Deep South	12	58	28	Geographic
9GE901	1520	1580	Southeast NA	Deep South	5	11	4	Geographic
9GE903	1300	1600	Southeast NA	Deep South	12	11	102	Geographic
Dyar	1375	1580	Southeast NA	Deep South	5	56	54	Plaza
Hickory Log	-150	1350	Southeast NA	Deep South	16	39	6	Geographic
Rucker's Bottom	1100	1450	Southeast NA	Deep South	6	37	62	Plaza
Town Creek	1350	1450	Southeast NA	Deep South	4	59	67	Mound
Town Creek	1150	1350	Southeast NA	Deep South	10	59	46	Plaza
Bessemer	1000	1200	Southeast NA	Deep South	4	20	63	Plaza
Chota-Tanase HI	1625	1800	Southeast NA	Deep South	27	39	89	Mound
Dallas	1300	1525	Southeast NA	Deep South	23	32	13	Mound
Hiwassee Island	1100	1300	Southeast NA	Deep South	11	41	58	center of plaza
King	1450	1625	Southeast NA	Deep South	43	55	57	Central Post
Martin Farm	1300	1600	Southeast NA	Deep South	2	65	44	Geographic

Site	Begin Date	End Date	Region	Subregion	Measured Houses	Median Size (m2)	Median Distance (m)	Central Place
Martin Farm	1000	1300	Southeast NA	Deep South	3	23	47	Geographic (between mound 1 and 2)
Martin Farm	900	1000	Southeast NA	Deep South	1	24	20	Mound 1
Mialoquo	1760	1776	Southeast NA	Deep South	7	33	41	Plaza
Rymer	1450	1575	Southeast NA	Deep South	23	41	31	Geographic
Moundville	1150	1300	Southeast NA	Deep South	3	40	300	Mound A
Kincaid	1000	1450	Southeast NA	Midwest	23	30	125	Plaza
SunWatch	1000	1500	Southeast NA	Midwest	17	39	51	Central Post
Yangoru Boiken, Sima village	1979	1981	Melanesia	New Guinea	143	38	257	Meeting Place
Haneo'o	1000	1800	Polynesia	Hawaiian Islands	3	15	1114	natural
Honokalani	1000	1800	Polynesia	Hawaiian Islands	23	21	39	natural
Kawaipapa	1000	1800	Polynesia	Hawaiian Islands	15	43	1118	natural
Wakiu	1000	1800	Polynesia	Hawaiian Islands	12	50	99	natural
Mohopilo	1000	1800	Polynesia	Hawaiian Islands	130	12	697	natural
Keahuolu	1000	1800	Polynesia	Hawaiian Islands	101	8	130	natural
Kawaihai 2	1000	1800	Polynesia	Hawaiian Islands	92	22	316	natural
Pahoehoe	1000	1800	Polynesia	Hawaiian Islands	59	40	65	natural
Matakawau Pa / Stingray Point Pa	1250	1800	Polynesia	New Zealand	37	110	53	highest feature (tihi)
Ngaungau Pa / Poor Hill	1250	1800	Polynesia	New Zealand	42	50	59	highest feature (tihi)
Poutukiterangi Pa	1250	1800	Polynesia	New Zealand	79	49	115	highest feature (tihi)
Maungarei Pa/ Mt Wellington Pa	1250	1800	Polynesia	New Zealand	113	142	175	highest feature (tihi)
Maungawhau Pa / Mt Eden Pa	1250	1800	Polynesia	New Zealand	87	180	244	highest feature (tihi)

Site	Begin Date	End Date	Region	Subregion	Measured Houses	Median Size (m2)	Median Distance (m)	Central Place
Ohinerau Pa / Mt Hobson Pa	1250	1800	Polynesia	New Zealand	55	266	99	highest feature (tihi)
Titikopuke Pa / Mt St John Pa	1250	1800	Polynesia	New Zealand	33	175	53	highest feature (tihi)
Pa	1250	1800	Polynesia	New Zealand	9	51	14	highest feature (tihi)
Hitiuira	1200	1800	Polynesia	Rapa Nui	78	7	556	natural
Huanuco Pampa	1450	1532	Central Andes	Peruvian Highlands	272	560	385	Ushnu
Agua Rica	1300	1550	Southern Andes	Carangas	32	9	112	ceremonial place
Tastil	1300	1450	Southern Andes	NW-Argentina	211	166	114	central plaza
Huanopatapampa	1200	1400	Southern Andes	Southern Altiplano	62	122	158	central area
Jach'a Pucara	1200	1450	Southern Andes	Southern Altiplano	111	178	103	Central plaza
Loma Bajala	1150	1450	Southern Andes	Southern Altiplano	66	172	59	Central plaza
Sivingani	1200	1450	Southern Andes	Southern Altiplano	13	123	91	Supra-local storage sector (gate)

References Cited

1. Johnson AW & Earle T (1987) *The Evolution of Human Societies: From Foraging Group to Agrarian State* (Stanford University Press, Stanford).
2. Feinman GM & Carballo DM (2022) Communication, Computation, and Governance: A Multiscalar Vantage on the Prehispanic Mesoamerican World. *Journal of Social Computing* 3(1):91-118.
3. Bogaard A, Bowles S, & Fochesato M (2019) The farming-inequality nexus: new insights from ancient Western Eurasia. *Antiquity* 93(371):1129-1143.
4. Drennan RD, Berrey CA, & Peterson CE (2015) *Regional Settlement Demography in Archaeology* (Eliot Werner Publications, Bristol, CT).
5. Leyk S*, et al.* (2020) Two centuries of settlement and urban development in the United States. *Science Advances* 6(23):eaba2937.
